# Metric Learning in Freewill EEG Pre-Movement and Movement Intention Classification for Brain Machine Interfaces

**DOI:** 10.3389/fnhum.2022.902183

**Published:** 2022-07-01

**Authors:** William Plucknett, Luis G. Sanchez Giraldo, Jihye Bae

**Affiliations:** Department of Electrical and Computer Engineering, University of Kentucky, Lexington, KY, United States

**Keywords:** Electroencephalogram (EEG), brain machine interfaces (BMIs), information theoretic learning (ITL), metric learning (ML), support vector machine (SVM), movement intention decoding, pre-movement intention decoding, readiness potential (RP)

## Abstract

Decoding movement related intentions is a key step to implement BMIs. Decoding EEG has been challenging due to its low spatial resolution and signal to noise ratio. Metric learning allows finding a representation of data in a way that captures a desired notion of similarity between data points. In this study, we investigate how metric learning can help finding a representation of the data to efficiently classify EEG movement and pre-movement intentions. We evaluate the effectiveness of the obtained representation by comparing classification the performance of a Support Vector Machine (SVM) as a classifier when trained on the original representation, called Euclidean, and representations obtained with three different metric learning algorithms, including Conditional Entropy Metric Learning (CEML), Neighborhood Component Analysis (NCA), and the Entropy Gap Metric Learning (EGML) algorithms. We examine different types of features, such as time and frequency components, which input to the metric learning algorithm, and both linear and non-linear SVM are applied to compare the classification accuracies on a publicly available EEG data set for two subjects (Subject B and C). Although metric learning algorithms do not increase the classification accuracies, their interpretability using an *importance* measure we define here, helps understanding data organization and how much each EEG channel contributes to the classification. In addition, among the metric learning algorithms we investigated, EGML shows the most robust performance due to its ability to compensate for differences in scale and correlations among variables. Furthermore, from the observed variations of the *importance* maps on the scalp and the classification accuracy, selecting an appropriate feature such as clipping the frequency range has a significant effect on the outcome of metric learning and subsequent classification. In our case, reducing the range of the frequency components to 0–5 Hz shows the best interpretability in both Subject B and C and classification accuracy for Subject C. Our experiments support potential benefits of using metric learning algorithms by providing visual explanation of the data projections that explain the inter class separations, using *importance*. This visualizes the contribution of features that can be related to brain function.

## Introduction

Brain-machine interfaces (BMIs) allow individuals to interact with machines through direct interpretation of their brain signals. One of the main goals for developing BMIs has been improving the quality of life of patients with debilitating conditions such as paralysis (Chaudhary et al., 2016; Lebedev and Nicolelis, 2017). Different methods measuring neural activities have distinctive spatial and temporal resolutions which have direct influence on what and how information can be processed. Electroencephalogram (EEG) allows non-invasive recording of neuronal activities, with benefits of safety and ease relative to invasive recording procedures, including intracortical BMIs that require surgical procedures to implant multielectrode arrays inside of the brain (Abiri et al., 2019; Rashid et al., 2020).

A sensory-motor rhythm-based BMI is the most popular EEG-based BMI paradigm for controlling external devices based on motor imagery (Yuan and He, 2014). A notable fact is that this motor imagery is defined as kinesthetic movement of any body parts such as hands, feet, and tongue, not necessarily related to the corresponding movement (Abiri et al., 2019). For example, in a sensory-motor rhythm-based BMI, a right hand could be imagined controlling an external device toward the right direction, a left hand for left, a right foot for up, and a left foot for down directions. The imagination of the body parts helps to provide distinctive EEG patterns, overcoming the low spatial resolution and signal to noise ratio, so this approach has been popularly employed in EEG-based BMIs. This is reflected on publicly available EEG data sets for motor imagery that mostly cover sensory-motor rhythm-based BMI setups (Agarwal, 2020).

However, it should be noted that the discrepancies between the representation of the body parts and the actual movements of the external device require large amounts of calibration data and lengthy training periods for the participants ranging from several days to weeks. Moreover, this approach fails to follow a natural and intuitive way of controlling external devices (Kim et al., 2015). To accomplish the direct extraction of movement kinematics so that a subject can imagine the natural way of movement to control external devices, we may need to represent the EEG in a more appropriate way for neural decoding.

In addition, the time instant when the neural activity is decoded is an important aspect to design more realistic BMIs. Traditionally, BMIs have been focused on decoding motor imagery right after movement onset, since dominant primary motor activities can be detected after the movement onset (Yong and Menon, 2015; Kaya et al., 2018; Kim et al., 2019). However, decoding pre-movement intention, which is measured prior to movement onset, can help pursuing more realistic BMIs capable of complex tasks. Readiness potential, which is also called Bereitschaftspotential or pre-movement potential, is known as brain activity associated with movement preparation (Schurger et al., 2021). It has been reported that its early components can be observed around ~1.5–0.4 s prior to onset, and late components can be detected around ~0.4–0 s before the movement onset (Brunia et al., 1985; Damen et al., 1996; Rektor, 2000). The unique pattern of the readiness potential supports the possibility of classifying different pre-movement intentions using EEG signals occurring before the movement onset. EEGs associated with pre-movement have been used to classify movement direction in a four-target center-out reaching task (Kim et al., 2019) and to decode voluntary finger movement intention in key pressing (Wang et al., 2020). Moreover, pre-movement EEG correlations among upper limb analytic movements (López-Larraz et al., 2014) and complex upper limb movements (Mohseni et al., 2020) have been reported. It has also been shown that target objects could be predicted before movement from EEG in a virtual environment to choose one object from presenting two or three object options (Novak et al., 2013).

Although the possibilities of classifying EEG pre-movement intention have been reported, this is a challenging task and the interpretation of neural activities in different brain areas is still lacking. It is well-known that after the movement onset, EEG recorded from primary motor cortex (commonly from channel C3 and C4) shows distinctive EEG patterns to different movements, which helps to classify these intentions appropriately. However, relatively less dominant and complex neural activities prior to the movement onset are more challenging to detect (Andersen and Cui, 2009). Thus, previous research has been mainly focused on premotor cortex and primary motor cortex, emphasizing C3 and C4 channels in EEG. Methods that exploit the joint activity in multiple brain areas and that can capture relations beyond simple linear correlations have the potential advantage for extracting the information required to decode pre-movement intention.

Metric learning is one type of machine learning algorithm that finds a transformation that reorganizes data by optimizing an objective function. Metric learning can be understood as the problem of learning a representation, a mapping, of the data that yields some desired notion of similarity between data points (Bellet et al., 2015; Suárez et al., 2021). In other words, the objective function in metric learning focuses on how the machine learning model represents the data in terms of distances or similarities between data points. This is a distinctive aspect of metric learning which is conceptually different from learning a representation that directly optimizes classification performance. A representation learned for classification may impose structures, such as linear decision boundaries, to define a global rule that classifies data. This can result in ignoring informative features that are not linearly related to the class labels. In contrast, a representation learned to create a metric, where the notion of distance is relative to each data point, can be more flexible and be able to capture non-linear relations through local structures.

In this study, we explore metric learning techniques applied to the problem of learning a representation suitable for decoding pre-movement and movement intention in EEG-based BMIs. We focus on metric learning algorithms that linearly project data to a low dimensional space where neural activity corresponding to similar actions cluster together. The learned projections should not only be able to preserve class information, but also can highlight which EEG channels provide such information, adding interpretability to the learned representations. This unique feature of metric learning contrasts with methods purely based on classification performance which can be hard to interpret.

We study three metric learning algorithms whose objective function are motivated from different perspectives. The first algorithm is neighborhood component analysis (NCA) whose objective function is based on a proxy to k-nearest neighbor (k-nn) classification accuracy. The second and third algorithms considered are conditional entropy metric learning (CEML) and entropy gap metric learning (EGML) whose objective functions are based on information theoretic quantities. These choices are motivated by their potential to capture non-linear relations that are relevant for classification through a linear transformation to a low dimensional space. In NCA, the k-nn objective imposes very little structure on the classes allowing for local interactions between learned features. CEML and EGML can capture relations beyond simple correlations using conditional entropy and mutual information as descriptors of the data. CEML has been applied to the visualization of high-dimensional intracortical neural recordings from different tasks, by projecting them on 2-dimensional space (Brockmeier et al., 2013). It was observed that the obtained projection was able to capture the natural relations among different tasks using intracortical neural activity from non-human primates.

To the best of our knowledge, this is the first attempt to investigate metric learning algorithms and their interpretability in EEG pre-movement and movement intention classification. We expect this work will open the door to further investigation on the representation of pre-movement and movement intentions in EEG-based BMIs. In the following section, background on metric learning and support vector machines is provided. In Section Material and Methods, we describe which EEG data set we used, and how the EEG data set was organized to apply the metric learning algorithms. Details on the metric learning and classifier set ups are also provided. In the following section, our results and acknowledgment of the data are explained. At last, we conclude with potential effects and limitations of metric learning implementation on EEG pre-movement and movement intention classification.

## Background

### Metric Learning

In this section, we provide an overview of the metric learning algorithms applied to the EEG pre-movement and movement intention classification. A common approach for metric learning is to parametrize a distance function as a composition of a transformation, mapping, and the computation of a simple distance such the Euclidean distance between points after being transformed (Kulis, 2013). The parameters of the distance function are thus adjusted according to some desired behavior of the distance function. For instance, if we have access to class labels, we can train the model to find a distance that makes data points from the same class have small distances and points from different classes have large distances. In our setting, we define a linear mapping **A** from the original input space X, in our case, $X={\mathbb{R}}^{d}$, where *d* is the dimension of the input data, to a *p*-dimensional vector space ℝ^*p*^, where *p* is a free parameter. The distance *d*_**A**_ between data points **x**_*i*_ and **x**_*j*_ is given by


$$\begin{array}{l} d_{\text{A}}\left({\text{x}}_{i},{\text{x}}_{j}\right)=\|\text{A}\;{\text{x}}_{i}-\text{A}\;{\text{x}}_{j}\;\| \end{array}$$

where ‖·‖ is the Euclidean distance in ℝ^*p*^.

#### Euclidian Distance (Baseline Metric)

Euclidian distance is used as a baseline to understand the effect of the metric learning algorithms. That is, no metric learning is applied. In this case, the identity matrix is returned as the transformation matrix, that is, *d* = *p* and **A=I**. Classifiers are also trained using the Euclidean distance as the metric for representing the datapoints in the original, i.e., untransformed space X. We compare performances of Euclidian, CEML, EGML, and NCA.

#### Matrix-Based Conditional Entropy Metric Learning

CEML is an information theoretic learning technique that employs conditional entropy as an objective function to find a metric space where information about labels is maximally preserved. Like principal component analysis (PCA) and linear discriminant analysis (LDA), CEML reduces the dimensionality of the input, from *d* to *p*. However, unlike LDA, CEML makes less assumptions about how data is distributed. That is, CEML does not assume any type of data distribution, whereas LDA assumes the data classes follow a Gaussian distribution. Thus, LDA is not suitable when the data is not Gaussian distributed. In addition, since PCA is an unsupervised learning technique, no information about the class labels is used to derive the transformation.

For a set of *n* data, label pairs ${\left\{\left({\text{x}}_{i},\;l_{i}\right)\right\}}_{i=1}^{n}$, finding the representation that minimizes the conditional entropy given the projected data can be posed as the following optimization problem (Sanchez Giraldo and Principe, 2013):


(1) $$\begin{array}{l} \underset{\text{A}\;\in \;{\mathbb{R}}^{d\times p}}{\text{minimize}}\;S_{\alpha }\left(L|Y\right)\;,\text{subject to}{\;}_{\text{tr}\left({\text{A}}^{\text{T}}\text{A}\right)=p}^{{\text{A}}^{T}{\text{x}}_{i}={\text{y}}_{i},\;\text{for }i=1,\ldots ,n;\;} \end{array}$$

In this optimization problem, **A** is the parametrization of the linear transformation matrix by which the projected datapoints **y**_*i*_ are obtained from the original data points **x**_*i*_, where, ${\text{x}}_{i}\in {\mathbb{R}}^{d}$ and ${\text{y}}_{i}\in {\mathbb{R}}^{p}$ such that *p* ≪ *d*; *n* refers to the total number of exemplars. *S*_α_(*L*|*Y*) refers to the matrix-based conditional entropy of the labels *L*, given the projected datapoints *Y*, which is defined as the difference between the matrix-based joint and marginal entropies, *S*_α_(*L, Y*) and *S*_α_(*Y*), as follows:


(2) $$\begin{array}{l} S_{\alpha }\left(L|Y\right)=S_{\alpha }\left(L,Y\right)-S_{\alpha }\left(Y\right) \end{array}$$

The matrix-based entropy *S*_α_(*Y*) is an information theoretic quantity proposed in Sanchez Giraldo and Principe (2013), Sanchez Giraldo et al. (2015) that has similar properties to Rényi's α-order entropy but does not require the data distribution to be computed, and it is defined as


$$\begin{array}{l} S_{\alpha }\left(Y\right)=\frac{1}{1-\alpha }\log\left[tr\left({\left({\text{K}}_{Y}\right)}^{\alpha }\right)\;\right], \end{array}$$

where **K**_*Y*_ is a Gram matrix constructed by evaluating a positive definite kernel κ between all pairs of data points, that is, (**K**_*Y*_)_*ij*_**=**κ(**y**_*i*_, **y**_*j*_). The matrix-based joint entropy *S*_α_(*L, Y*) between random variables *L* and *Y* is defined as:


$$\begin{array}{l} S_{\alpha }\left(L,Y\right)=\frac{1}{1-\alpha }\log\left[tr\left({\left(\frac{K_{L}\circ K_{Y}}{tr(K_{L}\circ K_{Y})}\right)}^{\alpha }\right)\;\right], \end{array}$$

**K**_*L*_ **∘** **K**_*Y*_ is the Hadamard product between Gram matrices **K**_*L*_ and **K**_*Y*_. For *Y*, which takes values in ℝ^*p*^, a commonly used kernel is the Gaussian kernel,


$$\begin{array}{l} \kappa_{\sigma }\left({\text{y}}_{i},\;{\text{y}}_{j}\right)=\;\kappa_{\sigma }\left(\text{A}\;{\text{x}}_{i},\;\text{A}\;{\text{x}}_{j}\right)=\exp\left(-\frac{∥\text{A}\;{\text{x}}_{i}-\text{A}\;{\text{x}}_{j}{∥}^{2}}{2\sigma^{2}}\;\right) \end{array}$$

with σ > 0 as a scale parameter. For *L*, we use the correspondence (**K**_*L*_)_*ij*_ = 1 if **x**_*i*_ and **x**_*j*_ are in the same class and (**K**_*L*_)_*ij*_ = 0, otherwise.

The minimization part of Equation (1) refers to the minimization of the conditional entropy of the labels *L*, given the projected data points *Y***=A**^*T*^*X*. The first constraint ${\text{A}}^{T}{\text{x}}_{i}={\text{y}}_{i}\;$is necessary to maintain the relationship between the samples **x**_*i*_ in the original space and the transformed samples **y**_*i*_, and the trace constraint, *tr*(**A**^**T**^**A**) = *p*, is included to “avoid a trivial solution where the magnitude of **y**_*i*_ grows unbounded when the entries of **A** become large (Sanchez Giraldo and Principe, 2013).” α is a free parameter, α > 1. For all our experiments, we fixed the value of α = 1.01, which behaves similar to Shannon's entropy and has shown good empirical performance in other applications (Yu et al., 2020).

#### Entropy Gap Metric Learning

Finding a metric that minimizes the conditional entropy as defined above (Equation 2) is equivalent to maximizing mutual information defined as the difference between the matrix-based joint entropy and the sum of the marginal entropies, *S*_α_(*L, Y*) and *S*_α_(*L*) and *S*_α_(*Y*), as follows,


(3) $$\begin{array}{l} MI_{\alpha }\left(L;Y\right)={S_{\alpha }\left(L\right)+\;S_{\alpha }\left(Y\right)-\;S}_{\alpha }\left(L,Y\right) \end{array}$$

where *MI*_α_(*L*; *Y*) is the matrix-based mutual information of α order between the class label *L* and the projected data *Y***=A**^*T*^*X*. With this formulation, it is still necessary to control the scale of *Y*, by constraining the trace of **A**^*T*^**A**, to avoid trivial maximization of the mutual information since (Equation 3) would grow when we let **A** take large values. While the trace constraint keeps the objective function bounded, CEML is sensitive to the choice of the scale parameter σ of the kernel. Thus, selecting a proper σ must consider the range of the input data, which makes it challenging. To overcome this issue, we propose a notion of mutual information as the difference, *entropy gap*, between the expected value of the matrix-based joint entropy *S*_α_ (*L, Y*_Π_) that is obtained by randomly permuting the order of the samples with respect to the labels *L*, and *S*_α_ (*L, Y*), which is the joint entropy before the random permutation,


(4) $$\begin{array}{l} EG_{\alpha }\left(L;Y\right)=E_{\Pi }\left[S_{\alpha }\left(L,Y_{\Pi }\right)\right]-S_{\alpha }\left(L,Y\right) \end{array}$$

In other words, in Equation (4), we compute the difference between the average of the matrix-based joint entropies over randomly permuted data sets ${\left\{\left({\text{y}}_{\Pi_{i}},\;l_{i}\right)\right\}}_{i=1}^{n}$, where Π_*i*_ is a random permutation of the indices *i* = 1, …, *n*, and the matrix-based joint entropy of the original data set ${\left\{\left({\text{y}}_{i},\;l_{i}\right)\right\}}_{i=1}^{n}$. Unlike (Equation 3), where *MI*_α_(*L*; *Y*) increases as the values of **A** increase with a fixed kernel size parameter σ, the *entropy gap* (Equation 4) does not monotonically increase with increasing **A**. Instead, the *entropy gap* is only large for intermediate scaling factors and closes for small and large values relative to σ. More details about this property of the entropy gap are provided in the Supplementary Material. This behavior yields a way to automatically tune **A** to the scale that best captures the structure in the transformed data *Y***=A**^*T*^*X* that relates to the labels *L*. Note that we use the same α = 1.01 as CEML.

#### Neighborhood Component Analysis

The idea behind NCA is to provide a metric space for the data *X* that will perform well on a k-nn classifier (Goldberger et al., 2004). Optimizing the metric directly on the k-nn performance is difficult because this objective is highly discontinuous. Small variations in the data can change the nearest neighbors of a data point and therefore, change the class a point is assigned. Thus, the NCA algorithm approximates the leave one out error of a k-nn classifier by introducing a soft assignment where two points are neighbors with probability *P*_*ij*_. This probability is obtained based on the softmax function over the Euclidean distance of the transformed samples,


$$\begin{array}{l} P_{ij}=\frac{\exp\left(\|\text{A}\;{\text{x}}_{i}-\text{A}\;{\text{x}}_{j}{\|}^{2}\right)}{\sum_{i\neq k}^{n}\exp\left(\|\text{A}\;{\text{x}}_{i}-\text{A}\;{\text{x}}_{k}{\|}^{2}\;\right)} \end{array}$$

Based on this soft assignment, we can calculate the probability *P*_*i*_ that a point **x**_*i*_ is assigned correctly to its class *L*_*i*_ class as follows:


(5) $$\begin{array}{l} P_{i}=\underset{j\in L_{i}}{\sum }P_{ij} \end{array}$$

NCA finds the projection matrix **A** that maximizes the probability of correct classification for all points in the dataset,


$$\begin{array}{l} \underset{\text{A}\;\in \;{\mathbb{R}}^{d\times p}}{\text{minimize}}\;\overset{n}{\underset{i=\;1}{\sum }}P_{i} \end{array}$$

Since this function (Equation 5) is differentiable, NCA employs gradient descent for the parameter search.

### Classifier: Support Vector Machine

SVM is a discriminative learning approach that aims to find separating hyperplanes that are maximally far apart from the data points from each class (Alpaydin and Bach, 2014). One of the formulations for the linear version of the soft margin SVM is known as the ν-SVM (Schölkopf et al., 2000), where hyperparameter ν controls the percentage of margin errors and support vectors which indirectly trades off misclassification cost, and the regularization penalty on the decision hyperplane, the margin. We choose this formulation over the original formulation known as *C*-SVM (Cortes and Vapnik, 1995; Chang and Lin, 2011), which directly trades off between large margin and low classification error, because the ν parameter can be easier to set compared to *C* in *C*-SVM. Training the ν-SVM can be accomplished by solving the following quadratic problem:


$$\begin{array}{l} \underset{w,b,\xi ,\;\rho }{minimize}\frac{1}{2}\left\|w\right\|-\nu \rho +\frac{1}{n}\sum_{i=\;1}^{n}{\left(\xi \right)}_{i} \end{array}$$

(6) $$\begin{array}{l} subject\;to\;l_{i}\left({\text{w}}^{T}{\text{x}}_{i}+b\right)\geq \rho -{\left(\xi \right)}_{i},\;and\;\xi \geq 0,\;\rho \geq 0 \end{array}$$

where *l*_*i*_ ∈ {−1, 1}, indicating the class of data point **x**_*i*_. The non-linear version of this algorithm is based on the dual formulation of Equation (6) where the dot products are replaced with a positive definite kernel. The resulting decision function takes the form:


$$\begin{array}{l} f\left(\text{x}\right)=\;sign\left(\overset{n}{\underset{i=1}{\sum }}\beta_{i}\kappa_{SVM}\left(\text{x},\;{\text{x}}_{i}\right)+b\;\right) \end{array}$$

where *sign*(·) denotes the sign function *sign*(*z*) = 1 if *z* > 0 and *sign*(*z*) = −1 if *z* < 0, and κ_*SVM*_ is a kernel function, where we employ a Gaussian kernel,


$$\begin{array}{l} \kappa_{SVM}\left(\text{x},\;{\text{x}}_{i}\right)=\exp\left(-\frac{∥\text{x}-{\text{x}}_{i}{∥}^{2}}{2\sigma_{SVM}^{2}}\;\right) \end{array}$$

To distinguish this kernel size from the one used in metric learning, we denote it as σ_*SVM*_ here. The decision function in the non-linear case is more flexible. However, the input dimensions that influence the classification cannot be obtained directly from the learned parameters β_*i*_. For ν-SVM algorithm, both linear and non-linear, the value of ν must be selected and for the non-linear case, the parameters of the kernel function, σ_*SVM*_, must be tuned as well.

## Materials and Methods

### EEG Data

An EEG data set, which is freely available to the public, is used in this study. Details on the EEG acquisition set up and task paradigm can be found in Kaya et al. (2018). When a healthy adult participant is seating on a recliner chair with an EEG cap on their head, a computer screen, which was located ~200 cm in front at slightly above their eye level, presented a graphical user interface. A standard 10/20 international configuration was used for the EEG cap, with 19 bridge electrodes measuring scalp EEG. Additional two electrodes (A1 and A2) were placed at the earbuds as electrical ground. The EEG signal was sampled at a rate of 200 Hz, and a band-pass filter of 0.53–70 Hz and a 50 Hz notch filter were applied to the EEG data. All available data went through both filtering processes, and bad trials were already removed by the authors in Kaya et al. (2018). Thus, we did not apply any further pre-processing steps for the EEG data set.

We used a specific experiment paradigm called FreeForm in Kaya et al. (2018). To the best of our knowledge, this data set is the only publicly available EEG data set that is recorded for freely chosen target by the participants' own will and provides sufficient timeline for the pre-movement (i.e., prior to the movement onset) analysis. During the EEG recording session, the computer screen displayed a fixation point for participants to focus their attention upon during trials; the participants were asked to press either the “d” or “l” keys on the keyboard, using either their left or right hand. This experiment's unique set up is that the participants decided which key they will press at a given time, rather than being instructed to press a key determined by the experimenter. The 19 channel EEGs were recorded continuously throughout one recording session. One recording session contains ~700 trials. One trial corresponds to the participant pressing either the “d” or “l” keys. We represent **Class 1** as pressing “d” key and **Class 2** as pressing “l” key, and each class contains a balanced number of trials. We specifically used “Experiment FREEFORM-SubjectB-151111-2States,” “Experiment FREEFORM-SubjectC-151208-2States,” and “Experiment FREEFORM-SubjectC-151210-2States” files, and each file contains one session of EEG recording. We indicate **Subject B**, **Subject C Data 1**, and **Subject C Data 2** to refer “Experiment FREEFORM-SubjectB-151111-2States,” “Experiment FREEFORM-SubjectC-151208-2States,” and “Experiment FREEFORM-SubjectC-151210-2States” files, respectively. Information regarding which key was pressed was recorded in an extra channel at a synchronized time to the EEG data, and the key pressing time is considered as the movement onset.

**Figure 1:**
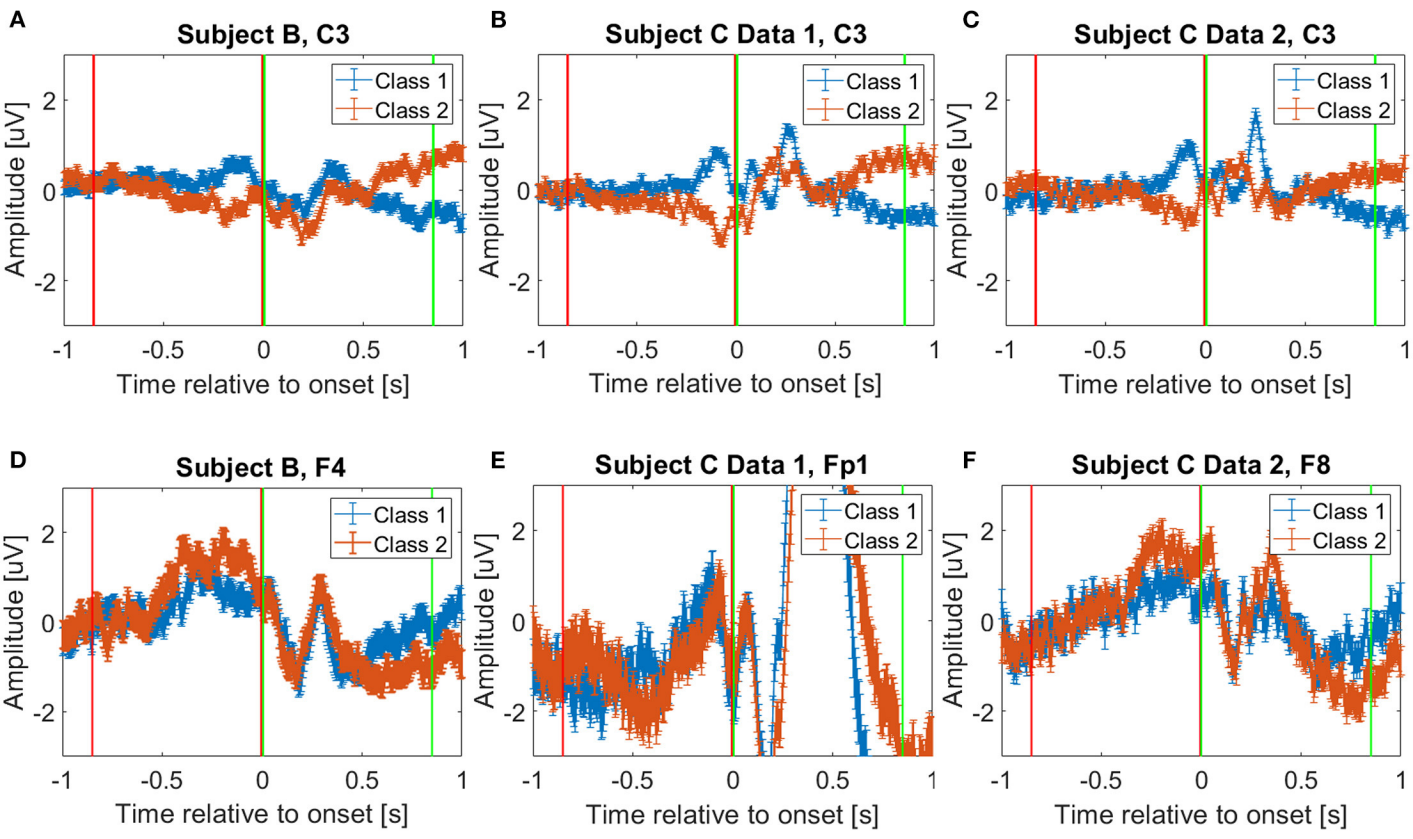
Average event related potentials for **(A)** Subject B on C3 channel, **(B)** Subject C Data 1 on C3 channel, **(C)** Subject C Data 2 on C3 channel, **(D)** Subject B on F4 channel, **(E)** Subject C Data 1 on Fp1 channel, and **(F)** Subject C Data 2 on F8 channel. Blue and orange solid lines show mean and standard error of the selected channels from trials corresponding to Class 1 and 2, respectively. The red and green vertical lines show where the pre-movement, [−0.85, 0] second interval, and movement intention, [0, 0.85] second interval, were extracted in this study, respectively. Note that *t* = 0 instant represents the movement onset when the “d” or “l” keys were pressed on the keyboard. We visualize C3 channel on **(A–C)** to provide fair comparisons with the previously reported study, which showed C3 channel deviations after the movement onset (Kaya et al., 2018).

To decode the pre-movement and movement intention, we extracted EEG for 0.85 s windows. For the movement intention, we used 0.85 s of EEG starting at the movement onset: i.e., [0, 0.85] second intervals, where we consider the movement onset as the time *t* = 0 point. We decided this specific starting time and window size based on a previously reported study for movement intention classification using the same data set (Mishchenko et al., 2019). Moreover, to investigate the pre-movement intention, we extracted 0.85 s EEG prior to the movement onset; that is, we used [−0.85, 0] second intervals, relative to the movement onset. Figure 1 shows the extracted time intervals for pre-movement (red vertical lines) and movement (green vertical lines) intention classification. The figure depicts challenges to distinguish different pre-movement intentions, compared to the clear distinction of the movement intention on the C3 channel (Figures 1A–C). However, we observe the potential to distinguish pre-movement intention from EEG amplitude differences in the frontal lobe (Figures 1D–F). F4, Fp1, and F8 channels are displayed for Subject B, Subject C Data 1, and Subject C Data 2, respectively. We chose these specific channels per data file since they show the most distinguishable amplitude differences in time during pre-movement intention in each data set. This phenomena can be explained by how brain organizes movement sequentially (Andersen and Cui, 2009). It is known that prefrontal and posterior cortex decide the movement, and primary motor cortex produce specific movements (Wise, 1985; Andersen and Cui, 2009).

### Pre-processing and Feature Representation

**Figure 2:**
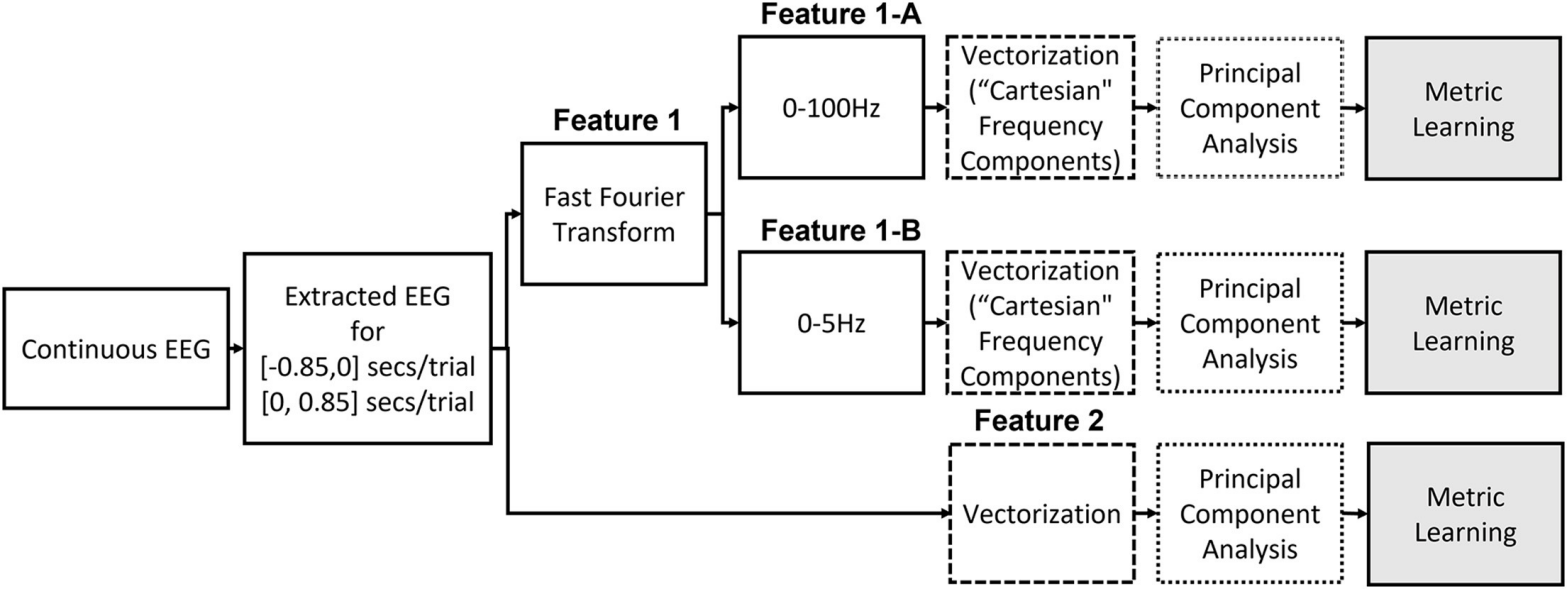
Illustration of feature extraction steps that include metric learning algorithms.

We consider two different features in this study, and Figure 2 illustrates the two different ways to construct the features from the raw EEG data.

#### Feature1: Selective Frequency Components

**Figure 3:**
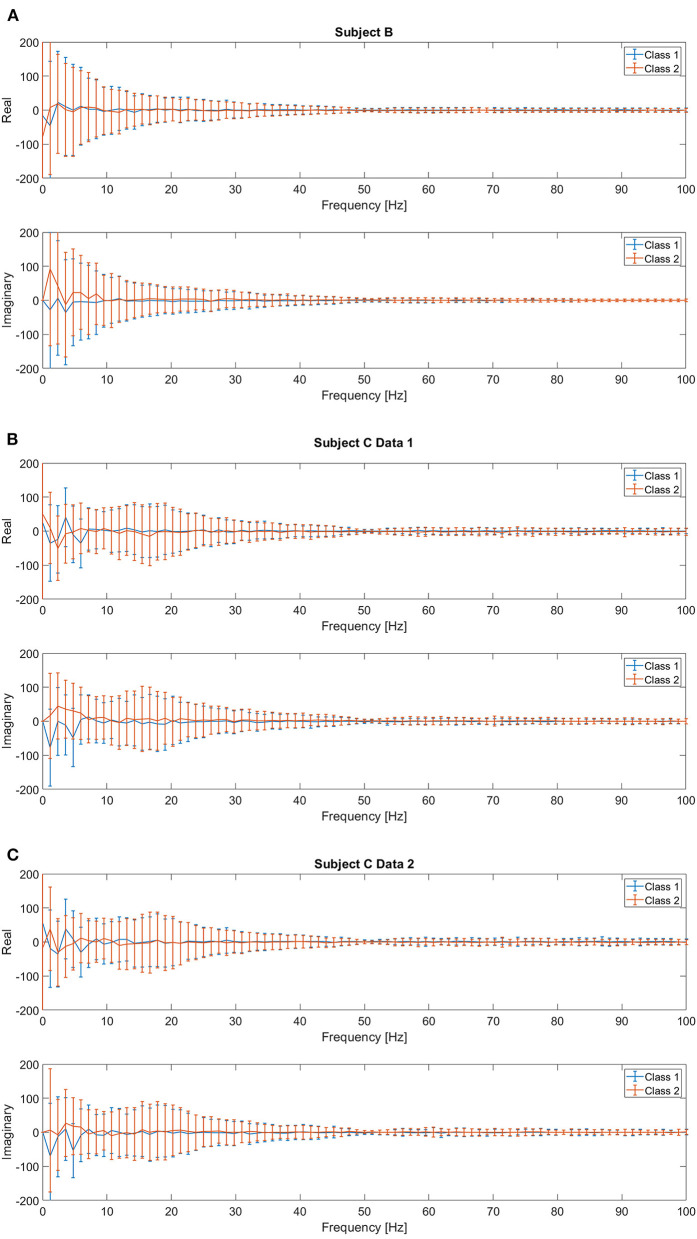
Mean and standard deviation of the real and imaginary values from fast Fourier transform for [0, 0.85] second intervals on C3 channel for **(A)** Subject B, **(B)** Subject C Data 1, and **(C)** Subject C Data 2.

Previous studies have reported that low frequency components extracted from the EEG signals followed by a linear SVM provide the best accuracy for movement intention classification (Kaya et al., 2018; Mishchenko et al., 2019). Therefore, we consider this feature and classifier as our baseline for comparison in this study. We followed the same procedure to construct the input features for both pre-movement and movement intentions. The details of feature extraction procedures can be found in Kaya et al. (2018). First, complex frequency components are obtained from the EEG window, *t* ∈ [−0.85, 0] seconds for pre-movement intention and *t* ∈ [0, 0.85] seconds for movement intention, for all 21 channels *via* fast Fourier transform. It was reported that using only frequency components from 0 to 5 Hz provides the best movement intention classification accuracy with a linear SVM for this data set (Kaya et al., 2018; Mishchenko et al., 2019). Since we additionally include the pre-movement intention classification problem, we consider 2 different configurations of the frequency ranges for comparisons: **Feature1-A**) all frequency components (0–100 Hz) and **Feature1-B**) only frequency components from 0 to up to 5 Hz. The complex frequency components were split into real and imaginary pairs for each frequency, and then, each channel's “Cartesian” Fourier transform values are concatenated into a single vector. Consequently, **Feature1-A** and **Feature1-B** produce a 3,549 (169 features × 21 channels) and a189 (9 features × 21 channels) dimensional vector per trial, respectively. Note that although **Feature1-A** has higher dimensionality (*d*=3,549) compared to **Feature1-B** (*d*=189), the data projection after metric learning (ℝ^*d*^ → ℝ^*p*^) yields a smaller dimensionality for the inputs to the SVM classifier. We set to investigate whether metric learning provides an effective organization of the data for EEG intention classification, even without preselecting the effective frequency components. Figure 3 shows the real and imaginary values of the resulting fast Fourier transform from channel C3 for the [0, 0.85] second interval. A clear difference between the values of the frequency components for two classes can be observed for frequencies <5 Hz, which supports the use of **Feature1-B**.

Note that (Mishchenko et al., 2019) investigated how much information is contained in different frequency ranges that contribute to EEG motor imagery classification tasks. This group used the same EEG data set as in Kaya et al. (2018), which is the data set we use in this study. They set the frequency range from 0 to 80 Hz, and then applied a filter with the following passing bands; 0–5, 5–10, 10–15, ···, and 75–80 Hz. It was reported that after ~20 Hz, the decoder's performance on the mental imagery classification dramatically decreased as the features include higher frequency components, and they concluded that the 0–5 Hz band showed the best performance. Although alpha or mu sensorimotor rhythms have been actively considered to classify EEG motor imagery, the use of slow potentials such as movement related cortical potentials has also been reported. In addition, Figure 3 supports that the low frequency components are the most distinctive in different intentions. Thus, we chose the 0–5 Hz range for **Feature 1-B**. In addition, we decided to include all frequency ranges (0–100 Hz with sampling frequency of 200 Hz) for comparison in **Feature 1-A**.

**Figure 4:**
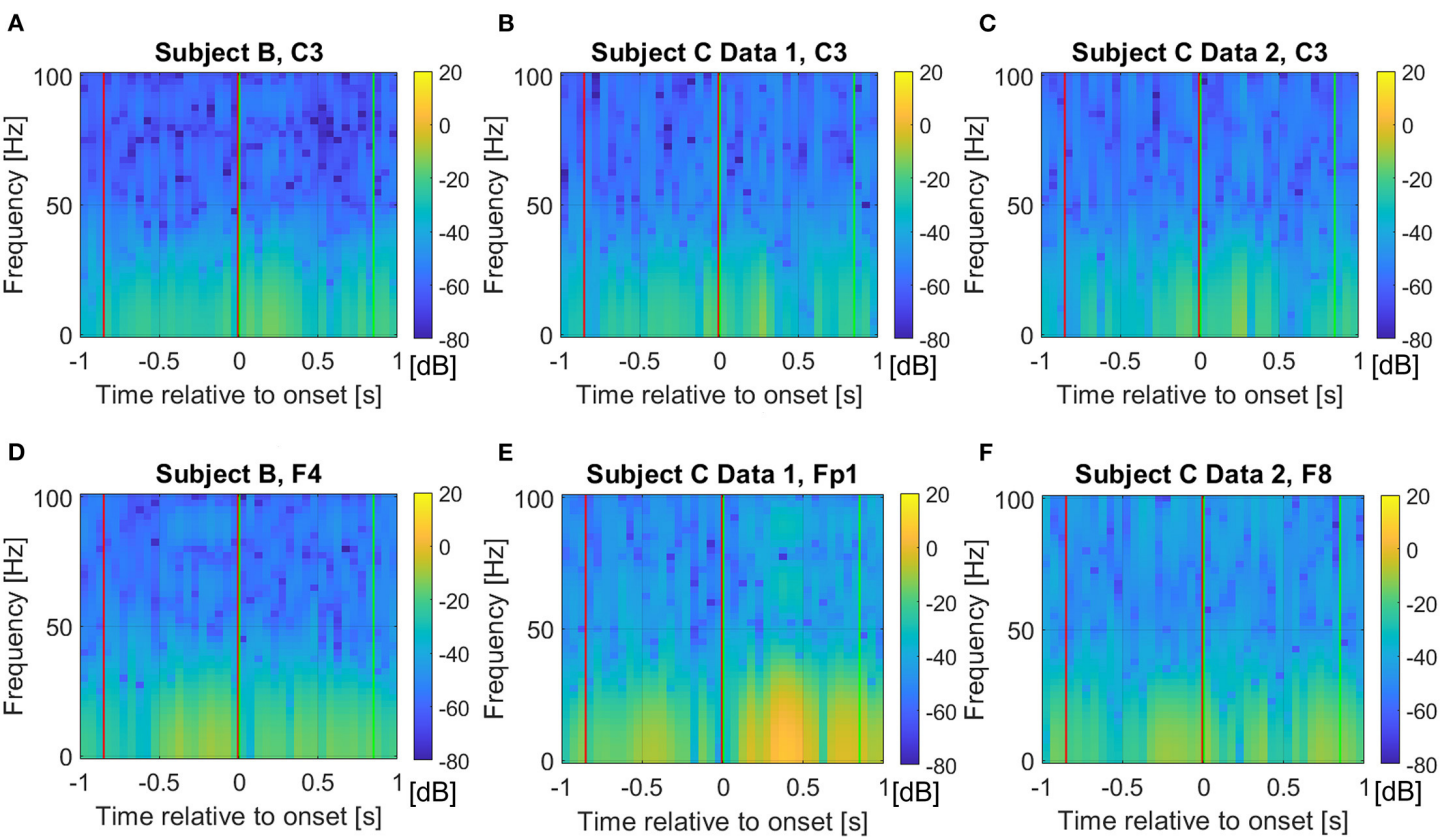
Average frequency component absolute differences in dB from the event related potentials between Class 1 and 2 for **(A)** Subject B on C3 channel, **(B)** Subject C Data 1 on C3 channel, **(C)** Subject C Data 2 on C3 channel, **(D)** Subject B on F4 channel, **(E)** Subject C Data 1 on Fp1 channel, and **(F)** Subject C Data 2 on F8 channel. The red and green vertical lines show where the pre-movement and movement intention were extracted in this study, respectively. We set the colormap limits from −80 to 20 dB for all cases for fair comparisons.

However, differences for higher frequency components are also observed, thus, we also investigate the full frequency range and raw time domain EEG, which are described in the following section. Figure 4 show the absolute frequency differences in dB, between the average event related potentials from Classes 1 and 2. As expected, based on Figures 1A–C, more distinctive frequency differences are observed for the signals that correspond to the “after movement” onset than for those prior to movement, on channel C3. However, as noted in Figures 1D–F, possible distinction before the movement onset is also observed in the selected channels (Figures 4D–F).

#### Feature2: Time Domain EEG

**Figure 5:**
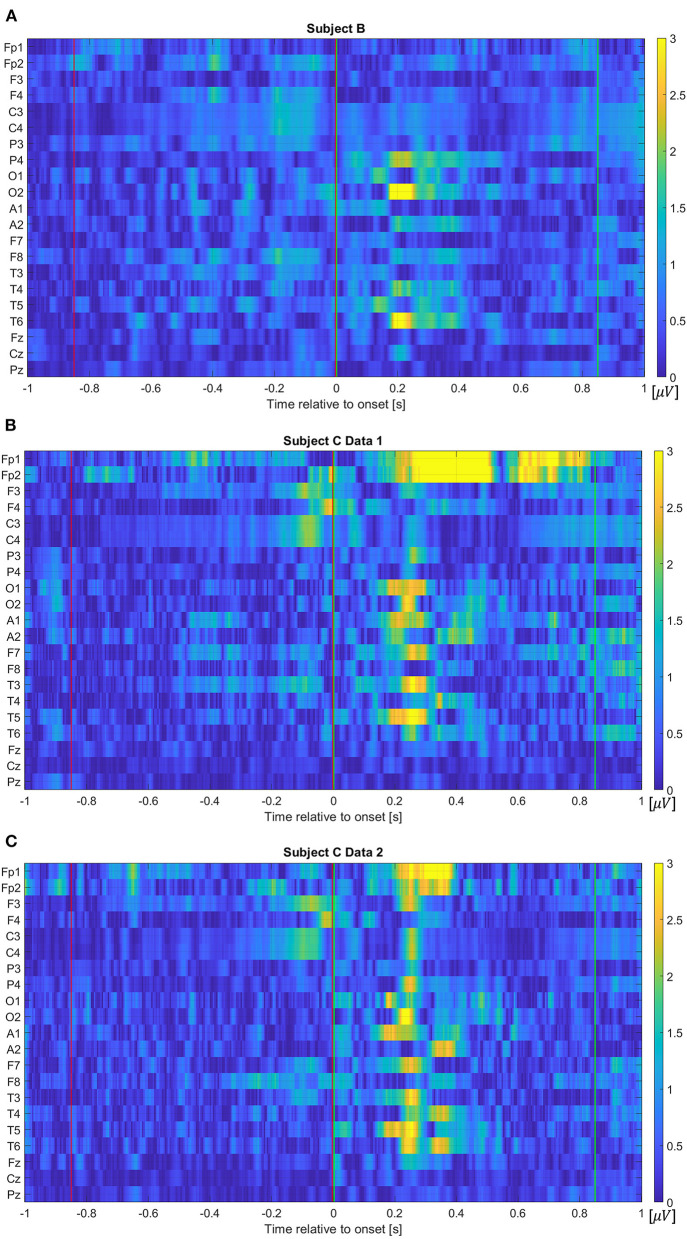
Absolute amplitude differences from average event related potentials between Class 1 and 2 for **(A)** Subject B, **(B)** Subject C Data 1, and **(C)** Subject C Data 2. The red and green vertical lines show where the pre-movement and movement intention were extracted in this study, respectively. Note that the color map values are forced to be limited within the range from 0 to 3 to evaluate pre-movement intention vividly. That is, values larger than 3 are displayed as yellow color due to the limit.

In addition to the frequency component features, we also investigate projected electrical potentials as features. To organize this feature, each channel's electrical potential, the time-series data, is first concatenated, which flattens the 2-dimensional data into a vector. The extracted EEG data for 0.85 s window contains 170 values per electrode due to the sampling frequency of 200 Hz, thus, the vectorized input dimension results in a 3,570-dimensional vector, when all 21 electrodes are considered. Figure 5 visualizes absolute amplitude differences from average event related potentials (ERPs) between Class 1 and 2 for each data set. As we have seen in Figure 1, more distinctive potentials are observed on all channels after the movement onset. However, although the difference is not as dominant as after the movement onset, electrical potential contrasts are noticeable before the movement onset.

#### Feature Conditioning *via* Principal Component Analysis

After features are vectorized, we applied principal component analysis (PCA) as a preprocessing step. This step reduces the input dimensionality below the number of available instances, which makes transformed data matrix have a rank equal to the number of PC dimensions, i.e., *d* = *d*_*pc*_. This provides better conditioning for the subsequent metric learning and classification stages and reduces the computational burden of their training. In addition, PCA can remove correlations due to the temporal structure of the data, potentially improving classification performance. We also normalized the total variance of the selected PCs, which is defined as the sum of the variances of all chosen dimensions, i.e., the trace of the covariance matrix in the PC space, to match the number of PCs, *d*_*pc*_. This normalization provides an expected range for the data which can be useful in setting the range for hyperparameter search, particularly the kernel size, σ for the metric learning algorithms and σ_*SVM*_ for the non-linear SVM classifier.

To select the best parameter configurations, we ran experiments with two different number of principal components. For **Feature1-A** and **Feature2**, we used *d*_*pc*_ = 300 and all non-zero variance PCs, which are at most the number of training samples, roughly *d*_*pc*_ = 560 in our case (for **Feature1-A** *d*=3,549 and for **Feature2** *d*=3,570). For **Feature1-B**, *d*_*pc*_ = 30 and all non-zero variance PCs, *d*_*pc*_ = *d* = 189. As we will see below, we use cross-validation to choose the optimal number of PCs *d*_*pc*_ in combination with metric learning dimension *p* and SVM parameters ν and σ_*SVM*_ for the non-linear case. Details of this procedure are provided in the following sections.

### Implementation of Metric Learning Algorithms and a Classifier

The metric learning algorithms are applied to the features (**Feature1-A**, **Feature1-B**, and **Feature2**) we described in the previous section. We applied a grid-search tuning for the metric learning algorithm's hyperparameters. We explored three different values for *p*, namely, 3, 10, and 100. Since PCA normalizes the data that serves as input to the metric learning algorithms, we fixed the kernel size for EGML and CEML to $\sigma =\sqrt{p/2}$. Finally, for both linear and non-linear SVMs, we search over ν ∈ {0.05, 0.1, 0.2, 0.3, 0.4, 0.5, 0.8}, and for the non-linear SVM, we search over kernel size parameters $\sigma_{SVM}\in \left\{\sqrt{5p},\sqrt{p},\;\sqrt{p/2},\;\sqrt{p/4},\;\sqrt{p/20}\right\}$. Test accuracies correspond to 10-fold cross-validation over the entire data set. For hyperparameter search, we ran a nested 10-fold cross-validation within each training partition, we call this partition the hyperparameter folds, for which all parameter configurations for PCA, metric learning, and SVM classification algorithms are trained. Based on the average among hyperparameter folds within a training partition, we choose the best combination of hyperparameters and test this configuration on the test fold.

Two classes are considered in the classification task. Classes 1 and 2 refer to trials pressing the “d” and “l” key, respectively. For the SVM implementation, we chose the widely used LIBSVM (Chang and Lin, 2011) library. Linear and non-linear SVMs are trained on the learned-metric.

#### Visualizing the Projections

**Figure 6:**
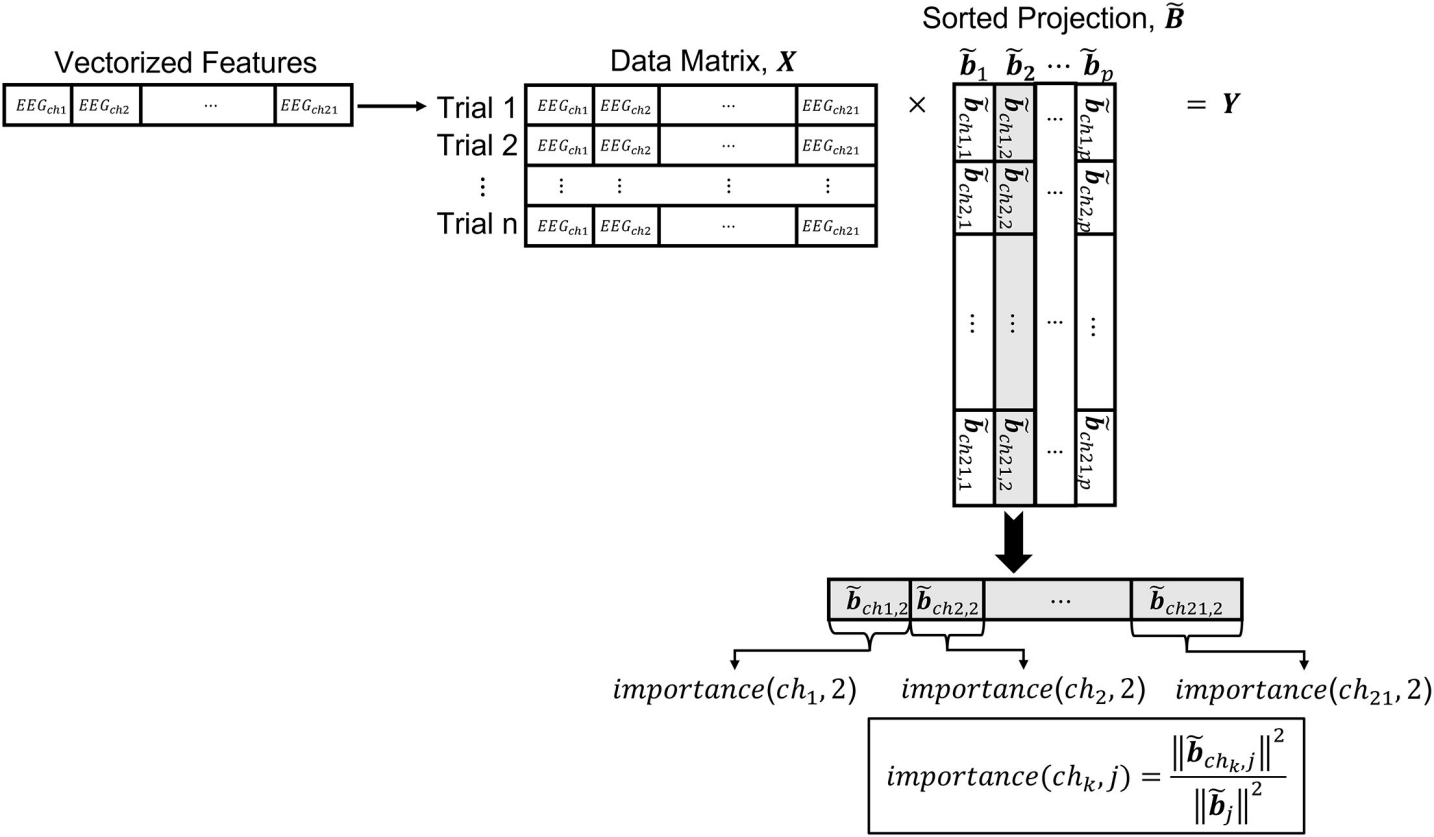
Illustration of data architecture to compute *importance*.

The metric obtained by transforming the input EEG features in ℝ^*d*^ to a lower dimensional space ℝ^*p*^ can be used to understand the contributions of the different EEG channels to the discrimination of neural states. Below, we describe a methodology to quantify the contribution of the inputs to the obtained metric. The EEG features that we analyze undergo a series of transformations. For instance, we apply PCA before metric learning. Therefore, to analyze the contribution of each one of the EEG channels to the discrimination between neural states, we need to include the PCA transformation as part of the projection. The overall transformation from the input data to the space learned by the metric, is a composition of several mappings. Namely, the projection matrix resulting from the composition of the PCA transformation matrix $\text{Q}\in {\mathbb{R}}^{d\times d_{pca}}$ and the matrix $\text{A}\in {\mathbb{R}}^{d_{pca}\times p}$ obtained from the metric learning algorithm that takes the selected PCs as input, is given by **B=QA**. Note that **B** ∈ ℝ^*d*×*p*^. Figure 6 illustrates a conceptual diagram to explain how the contribution of the input EEG channels, which we call here *importance*, is obtained from the transformations learned by the metric learning algorithms.

Since the metric in the projected space is invariant to any unitary transformation and shifts, we need to define a way to impose an order in the projected dimensions. We accomplish this by using the singular value decomposition of the projection matrix,


$$\begin{array}{l} \text{B}=\;\text{U}\text{D}{\text{V}}^{T}. \end{array}$$

where **U** and **V** are unitary transformations corresponding to the left and right singular vectors, and **D** is a diagonal matrix containing the singular values. We then define the sorted projection matrix


$$\begin{array}{l} \tilde{\text{B}}\;=\;\text{U}\text{D} \end{array}$$

where the singular values are ordered in descending order. The contribution of each input channel to each one of the learned features in the sorted projection matrix $\tilde{\text{B}}$ is obtained by taking the squared Euclidean norm of the entries in $\tilde{\text{B}}$ related to a channel and dividing it by the squared Euclidean norm of the entries for all channels combined. We call this measure *importance* (Equation 7). Let **x** ∈ ℝ^*d*^, be a vectorized sample from one of the EEG trials. For instance, if we are using **Feature1-B** (see Section Feature1: Selective Frequency Components), a vectorized sample **x** from one of the EEG trials would have *d* = *N*_*f*_ × *N*_*ch*_ = 189, where *N*_*f*_ is the number of frequency components in Cartesian form for each channel for 0~5 Hz and *N*_*ch*_ is the number of channels. For **Feature2**, a vectorized sample **x** from one of the EEG trials would have *d* = *N*_*t*_ × *N*_*ch*_ = 3, 570, where *N*_*t*_ is the number of time points of the window for the trial being considered. Then, for a sorted projection matrix $\tilde{\text{B}}\in {\mathbb{R}}^{d\times p}$ the *importance* of channel *ch*_*k*_ for feature in the projected space ℝ^*p*^ is given by:


(7) $$\begin{array}{l} importance\;\left(ch_{k},j\right)=\frac{\sum_{i\in I_{k}}{\left(\tilde{B}\right)}_{ij}{\;}^{2}}{\sum_{i^{\prime }=1}^{d}{\left(\tilde{B}\right)}_{i^{\prime }j}{\;}^{2}} \end{array}$$

Where *I*_*k*_ is the set of all input dimensions corresponding to channel *ch*_*k*_. For example, in **Feature1-B**, when *k* = 2, that is the channel is *ch*_2_, *I*_2_ = {10, 11, 12, 13, 14, 15, 16, 17, 18}. Note that the denominator of Equation (7) represents the squared Euclidean norm of the entire column of $\tilde{\text{B}}$, and the numerator is only for the entries corresponding to channel *ch*_*k*_. The overall *importance* of channel *ch*_*k*_ is given by


$$\begin{array}{l} \frac{1}{p}\overset{p}{\underset{j=1}{\sum }}importance\left(ch_{k}\;j\right) \end{array}$$

## Results

**Figure 7:**
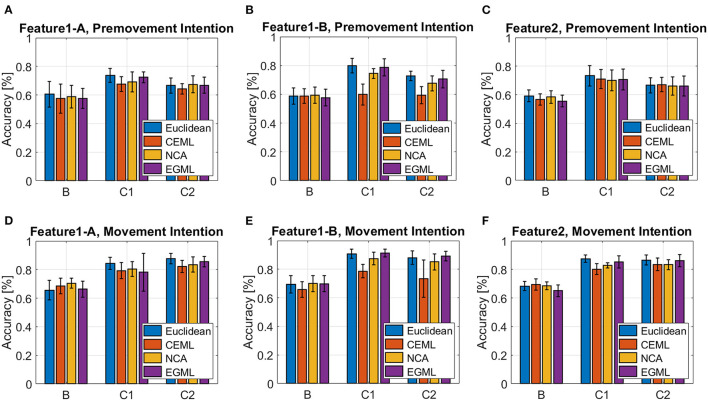
Classification accuracy for EEG pre-movement intention using **(A) Feature1-A, (B) Feature1-B**, and **(C) Feature2** and EEG movement intention using **(D) Feature1-A**, **(E) Feature1-B**, and **(F) Feature2**, when linear SVM was applied.

**Figure 8:**
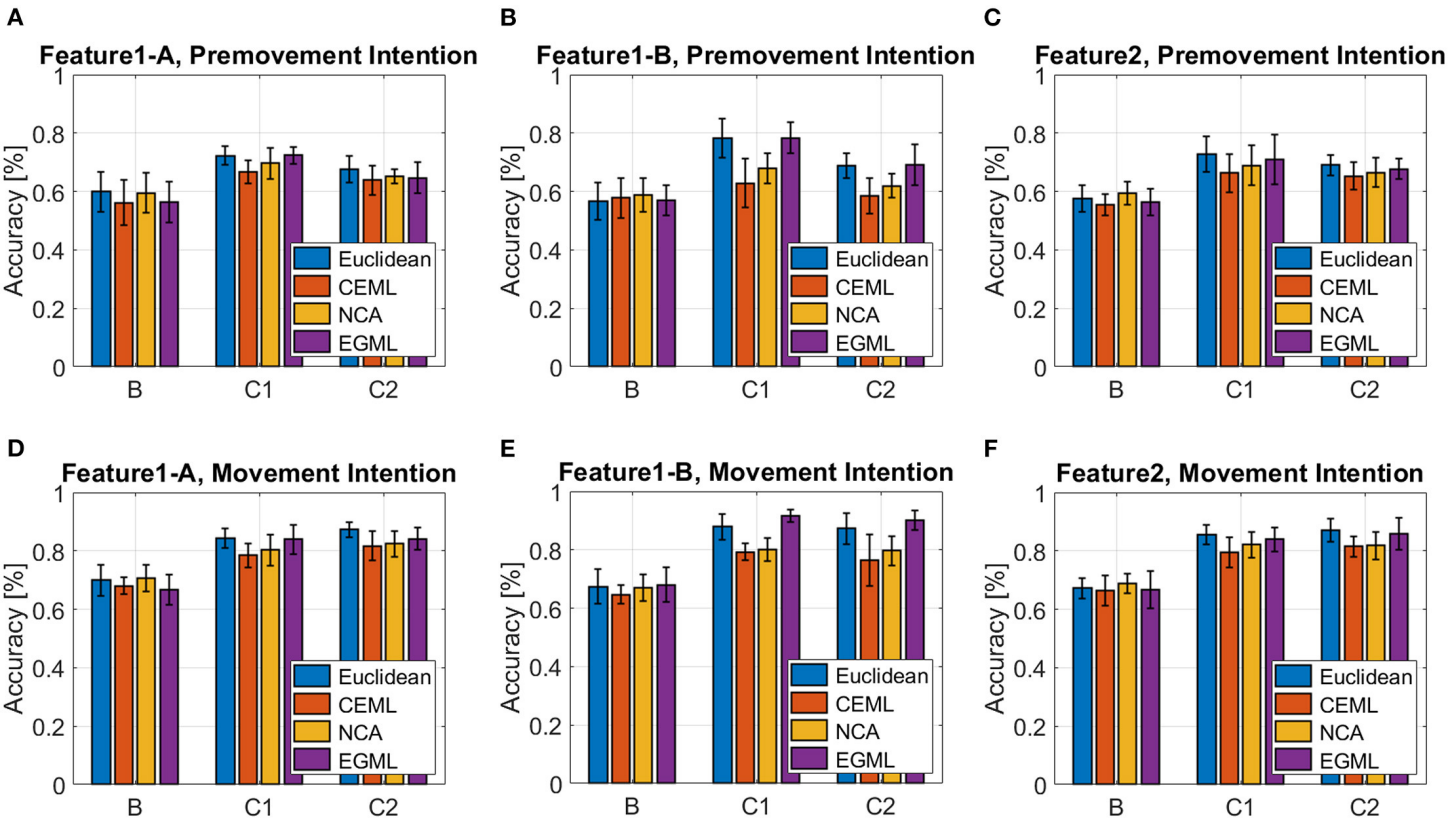
Classification accuracy for EEG pre-movement intention using **(A) Feature1-A, (B) Feature1-B**, and **(C) Feature2** and EEG movement intention using **(D) Feature1-A**, **(E) Feature1-B**, and **(F) Feature2**, when non-linear SVM was applied.

Figures 7, 8 showcase the performance of the classifiers to distinguish EEG pre-movement and movement intentions, with respect to the features we considered. Figure 7 displays results when linear SVM is applied to EEG intention classification, and Figure 8 shows results with non-linear SVM. Note that the linear and non-linear SVM classifiers are trained on the same data projections obtained by the metric learning algorithms, but the choice of the hyperparameters may differ based on the result of the cross-validation. We included the Euclidian (no metric is learned only PCA is applied) as a baseline. Our results on **Feature1-B** when using linear SVM match previously reported performance (Kaya et al., 2018). This was used as a sanity check for our implementation of EEG movement intention classification using **Feature1-B** (Figure 7E blue bars).

As we pointed out based on Figure 4 for **Feature1** and Figures 1, 5 for **Feature2** that show less distinctive patterns of EEG features, the pre-movement intention classification is more challenging. Relatively lower classification accuracies were observed as shown in Figures 7A–C, 8A–C compared to Figures 7D–F, 8D–F. In addition, lower performances were observed on Subject B compared to Subject C Data 1 (C1) and Subject C Data 2 (C2), due to the nature of different subject's EEG variability. This is a well-known challenge for obtaining a BCI neural decoder that generalizes well among subjects.

In addition, Subject B does not show significant performance variations over different features, whereas Subject C (C1 and C2) reveals variations on the performance over different metric learning algorithms depending on the employed features. In C1 and C2, all algorithms show similar performance on **Feature1-A** and **Feature2**, but variations when using **Feature1-B** are observed. Although **Feature1-B** provides the best classification accuracy in Subject C, **Feature2** shows reasonably good performance compared to **Feature1**. It is notable that **Feature2** only uses the raw EEG data in vectorized form, which can be extremely beneficial for the real-time implementation of BMIs. In addition, considering relatively similar electrical potentials between the two classes prior to the movement onset (Figure 1), the classification accuracy of the pre-movement intention using **Feature2** is impressive (Figures 7C, 8C). It is important to note that while classification accuracy does not improve after metric learning, the transformed data matches the accuracy of the Euclidean baseline but uses a much smaller number of dimensions. This means that the projected data captures the variations of the data that help distinguish between classes in a small subspace which becomes useful for the computation of *importance*.

Overall, EGML shows consistent performance over all features, and it provides the best matching classification accuracy to the Euclidean baseline, whereas CEML and NCA suffer more on the characteristics of the features. Paired *t*-test on the cross-validation test accuracies of the Euclidean baseline vs. EGML for movement intention using **Feature1-A** yield *p*-values 0.1552 (Supplementary Table 5), 0.4331 (Supplementary Table 6), and 0.8321 (Supplementary Table 7) for subject B, C Data 1 and C Data 2, respectively. For Euclidean vs. CEML *p*-values are 0.3436, 0.0039, and 0.0954, and for Euclidean vs. NCA 0.0509, 0.0283, and 0.5414 (Supplementary Tables 5–7). A complete set of tables with all comparison between different methods can be found in Supplementary Material (Supplementary Tables 5–13). When input dimensions have different variances, CEML and NCA tend to assign more weight to input dimensions with larger variance. CEML tendency for this is due to its trace constraint *tr*(**A**^**T**^**A**) = *p*, which is better suited for dimensions with equal variances. For NCA the convergence of its optimization algorithm is influenced by the input features with larger variances. Note that we applied the normalization to the total variance, the sum of all variances, rather than normalizing each dimension individually. Thus, further investigation of the *importance* maps focuses on the transformations obtained by NCA and EGML, only.

**Figure 9:**
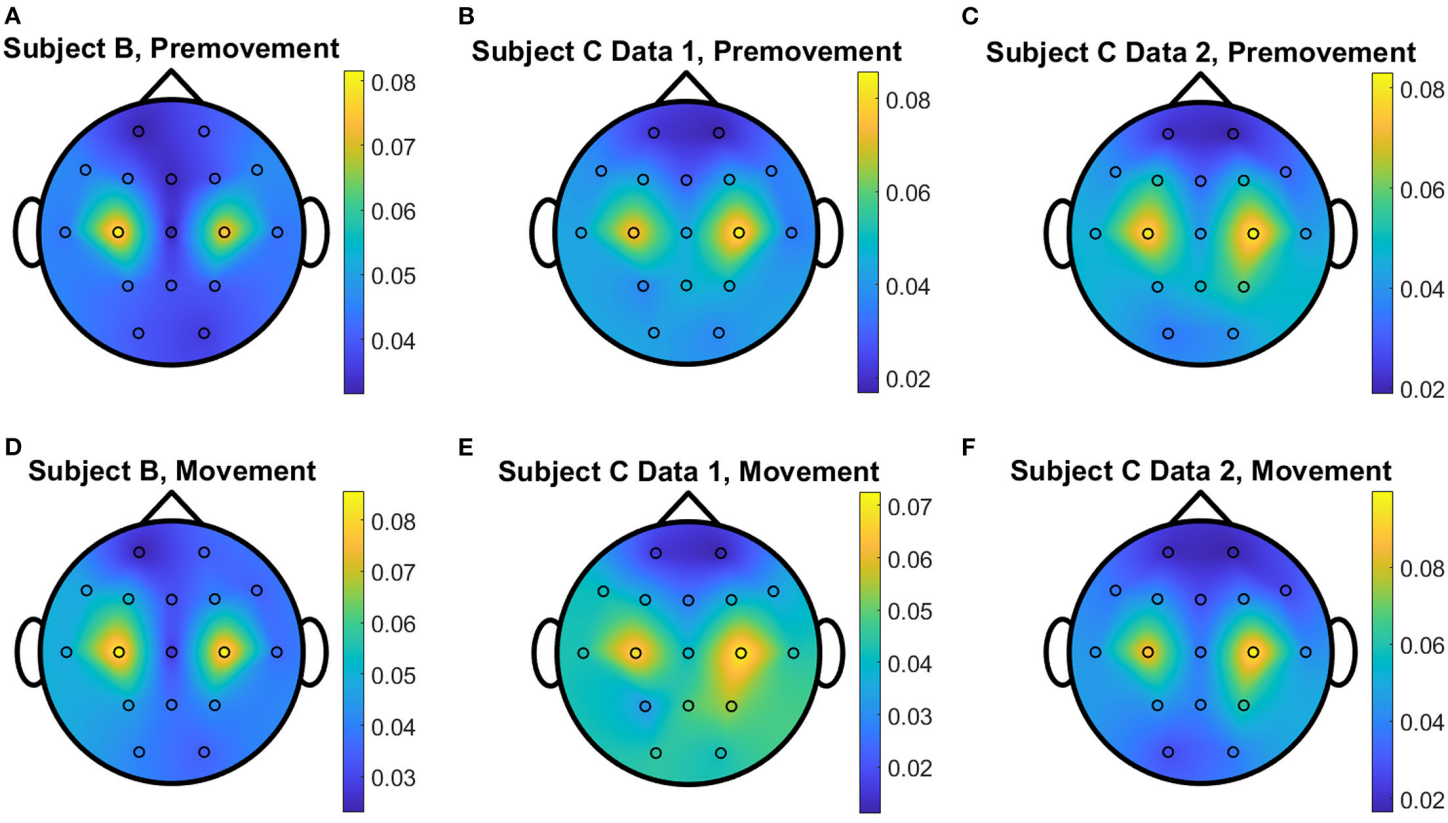
Conceptual topographical map, based on the extracted *importance* from metric learning, for EEG pre-movement intention classification for **(A)** Subject B, **(B)** Subject C Data 1, and **(C)** Subject C Data 2 and EEG movement intention classification for **(D)** Subject B, **(E)** Subject C Data 1, and **(F)** Subject C Data 2, when **Feature1-B** was used for EGML.

**Figure 10:**
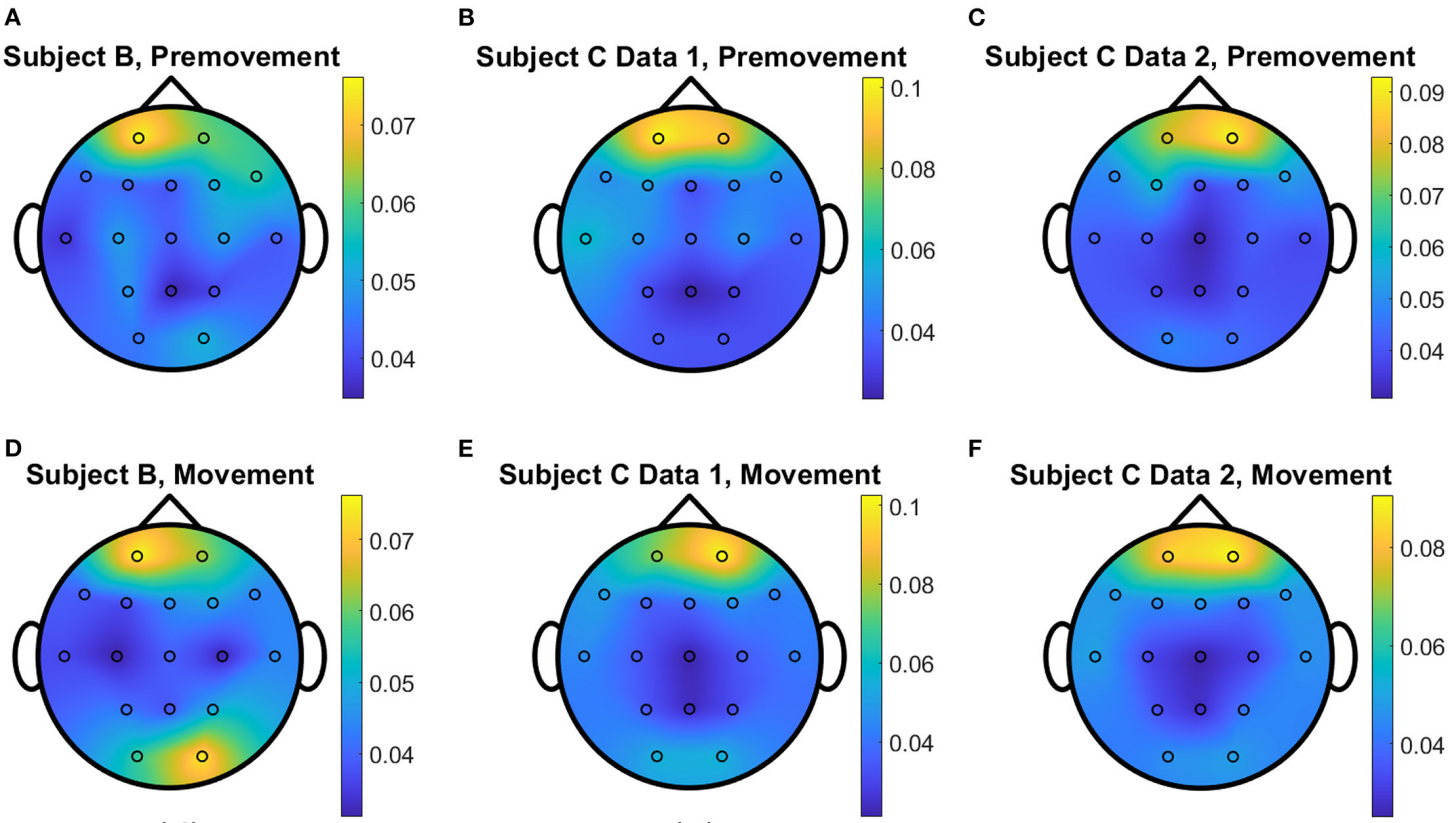
Conceptual topographical map, based on the extracted *importance* from metric learning, for EEG pre-movement intention classification for **(A)** Subject B, **(B)** Subject C Data 1, and **(C)** Subject C Data 2 and EEG movement intention classification for **(D)** Subject B, **(E)** Subject C Data 1, and **(F)** Subject C Data 2, when **Feature1-B** was used for NCA.

**Figure 11:**
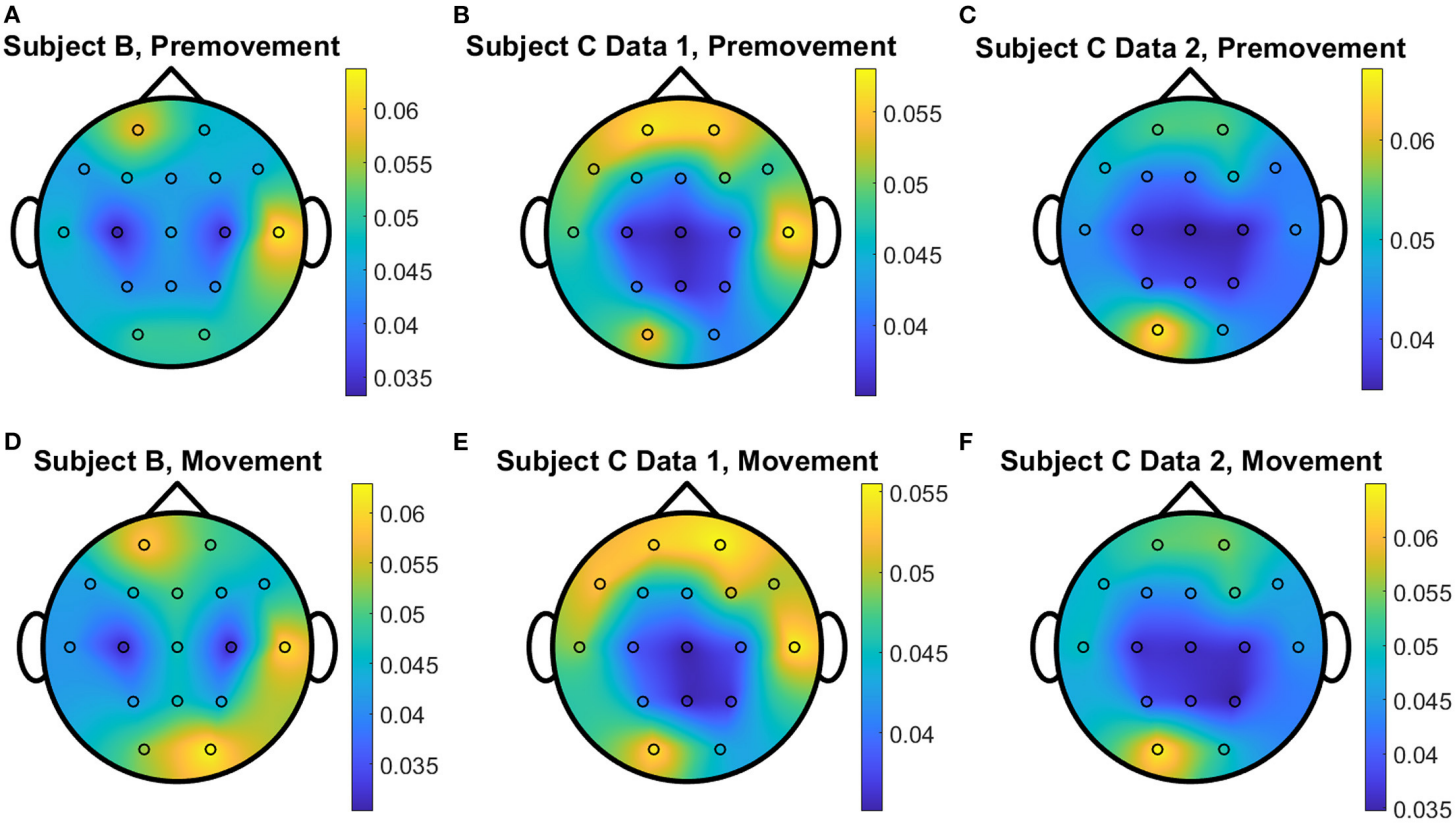
Conceptual topographical map, based on the extracted *importance* from metric learning, for EEG pre-movement intention classification for **(A)** Subject B, **(B)** Subject C Data 1, and **(C)** Subject C Data 2 and EEG movement intention classification for **(D)** Subject B, **(E)** Subject C Data 1, and **(F)** Subject C Data 2, when **Feature2** was used for EGML.

**Figure 12:**
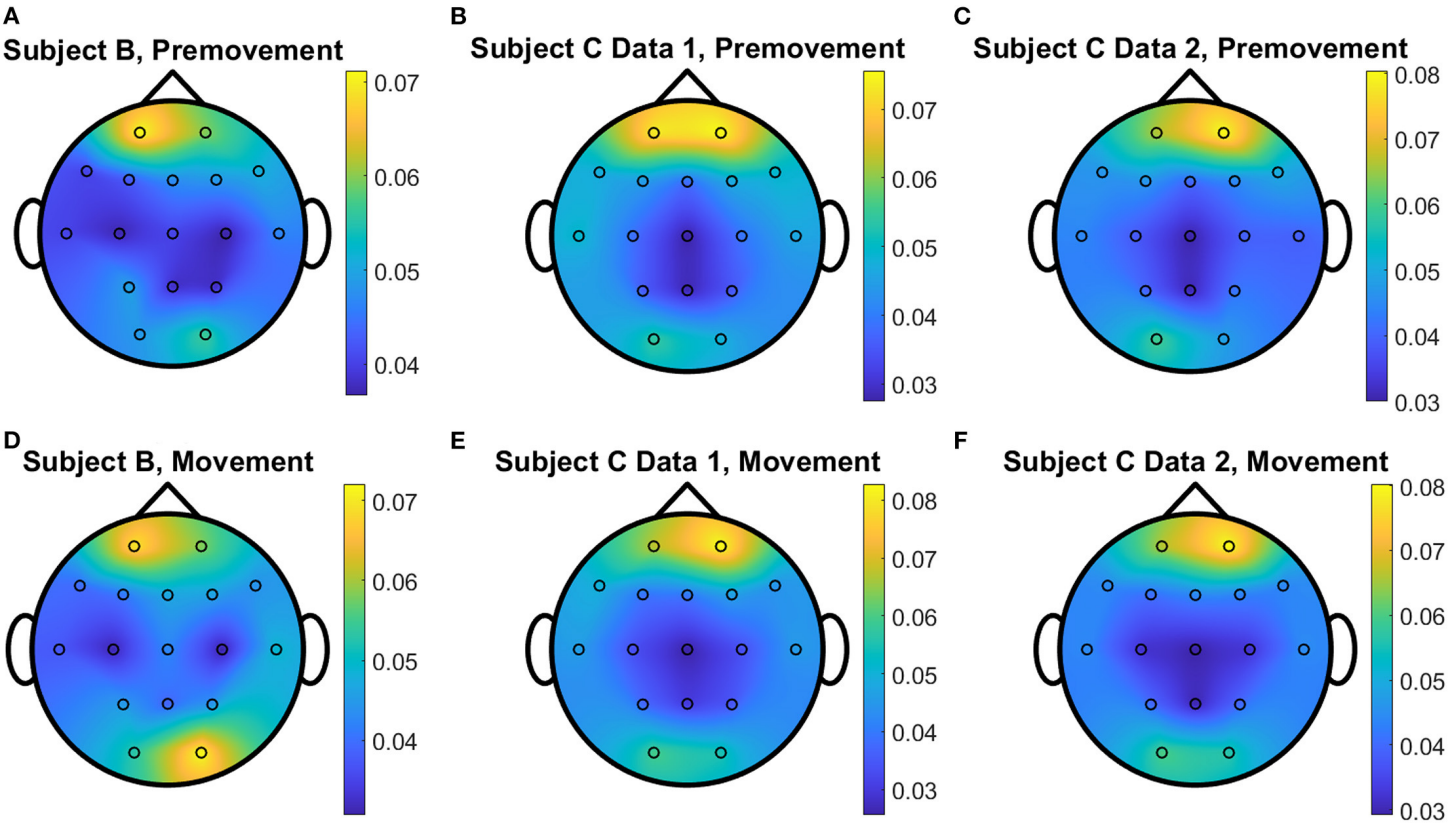
Conceptual topographical map, based on the extracted *importance* from metric learning, for EEG pre-movement intention classification for **(A)** Subject B, **(B)** Subject C Data 1, and **(C)** Subject C Data 2 and EEG movement intention classification for **(D)** Subject B, **(E)** Subject C Data 1, and **(F)** Subject C Data 2, when **Feature2** was used for NCA.

The overall distributions of *importance* for the EEG pre-movement and movement intention classification are displayed for each dataset as EEG topographic maps of the scalp. The average *importance* values over 10 folds and 3 top dimensions are displayed in Figures 9–12. When EGML is applied to **Feature1-B**, overall *importance* emphasizes on motor cortex for both pre-movement and movement intention (Figure 9), which supports the role of this cortical area in motor functioning (Rahnev et al., 2016; Firat, 2019). In contrast, the overall *importance* measured by NCA from **Feature1-B** emphasizes more on the frontal lobe (Figure 10), where more significant frequency component differences are observed compared to the ones from motor cortex (Figure 4). When **Feature2** is used, for movement intention, the focus occurs at the frontal and occipital lobes (Figures 11, 12), which coincides with the measured distance differences on the EEG after movement onset (Figure 5). The highly distinguishable occipital lobe's activity may be due to the visual stimulation in the experimental set up due to the screen display changing depending on which key is pressed. Note that **Feature1-A** contains Fourier transform components from the cropped EEG data which correspond to **Feature2**. Therefore, **Feature1-A** and **Feature2** have a linear relationship that causes the close to identical *importance* maps. In addition, interestingly, no remarkable differences on the average *importance* are observed between pre-movement and movement intention in all feature cases for both EGML and NCA. This may be due to the proximity to the onset point, *t* = 0, from both windows.

**Figure 13:**
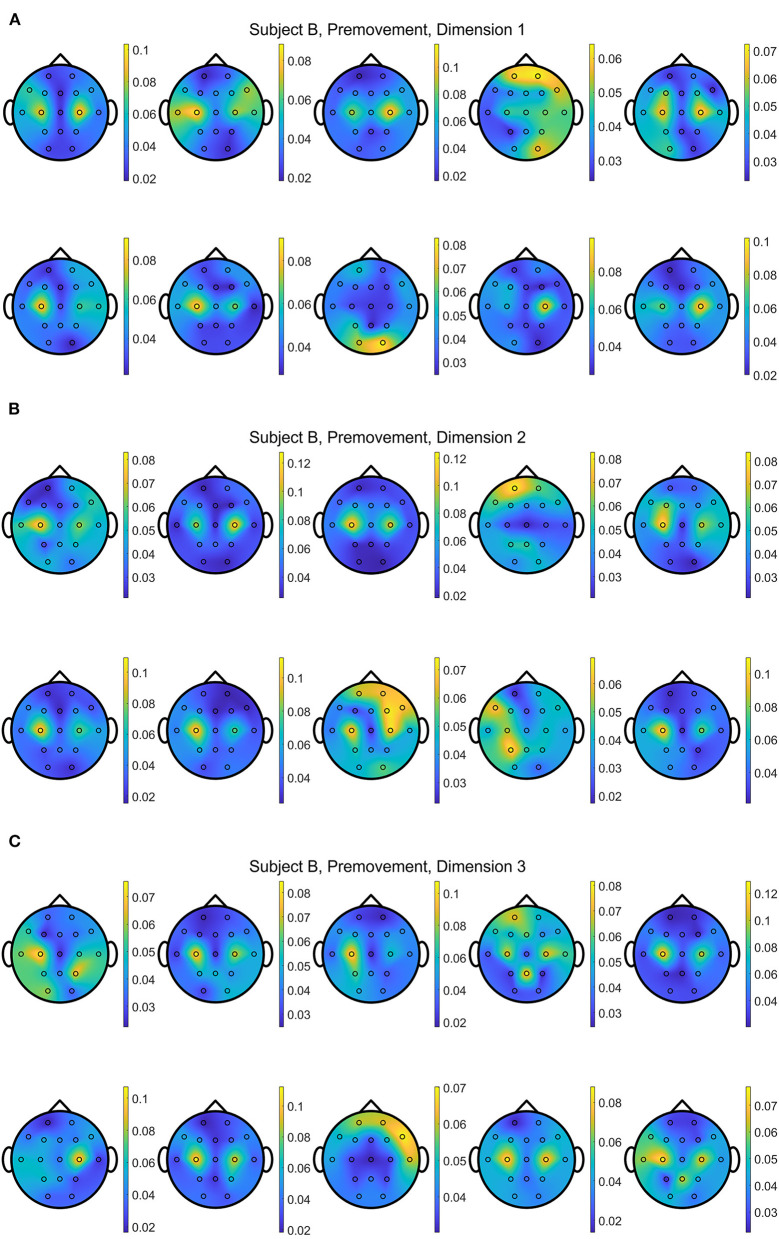
Conceptual topographical map, based on the extracted *importance* from metric learning, for EEG pre-movement intention classification for Subject B on **(A)** dimension 1, **(B)** dimension 2, and **(C)** dimension 3, when **Feature1-B** was used for EGML.

**Figure 14:**
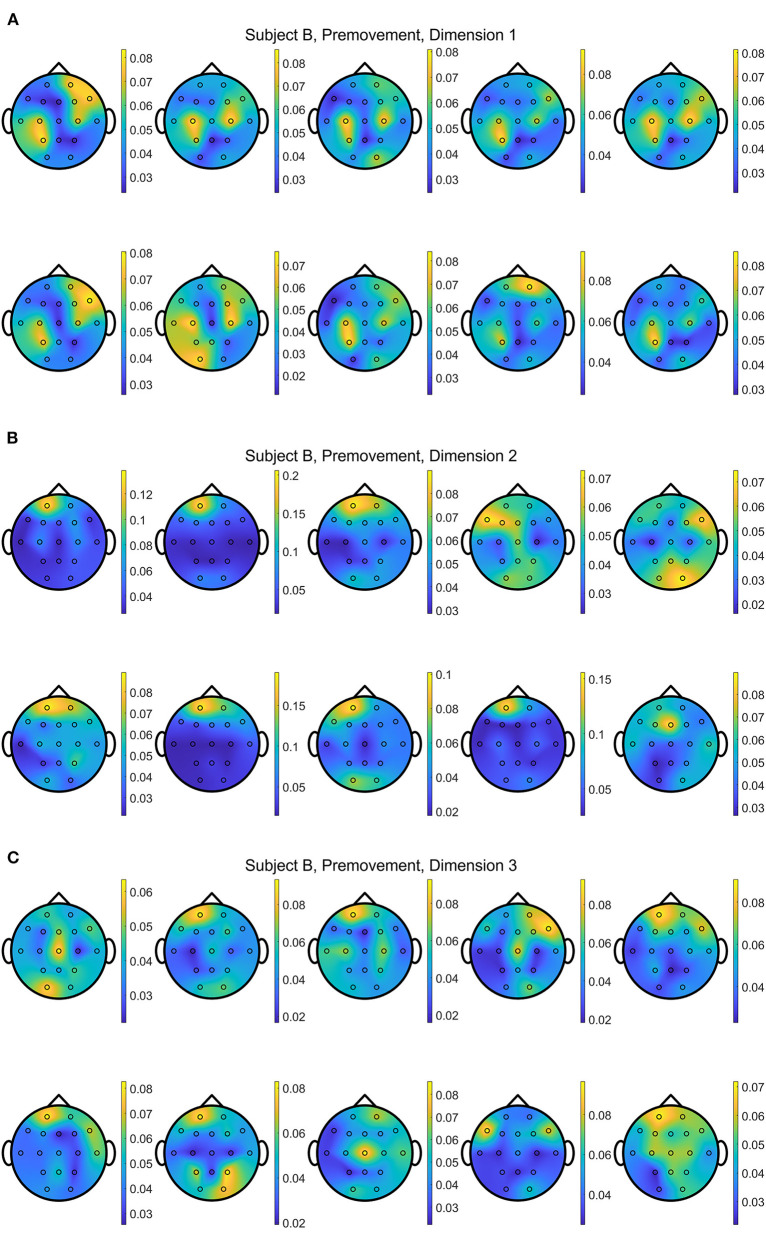
Conceptual topographical map, based on the extracted *importance* from metric learning, for EEG pre-movement intention classification for Subject B on **(A)** dimension 1, **(B)** dimension 2, and **(C)** dimension 3, when **Feature1-B** was used for NCA.

In addition, Figures 13, 14 show the distributions of *importance* for a data set with the selected metric learning algorithm over the projection dimensions 1, 2, and 3. We select Subject B for the visualization since this data shows clear focus on the motor cortex in C3 and C4 channels despite having the lowest classification accuracies compared to the other subject's data sets. This is a great example that supports the critical role of the metric learning algorithms in reorganizing the selected features to maximize the class differences in an interpretable fashion. Depending on the selected trials for the 10-fold cross validation, variations are observed. For example, some folds are mainly focused on the motor cortex on the 2nd or 3rd dimensions when EGML is used (Figure 13). However, when the *importance* maps over 10 folds and 3 dimensions are averaged, the key *importance* is clearly displayed. Figures 9A, 10A are the average of the 30 maps displayed in Figures 13, 14, respectively.

## Discussion and Future Directions

### Brain Functions in Movement and Interpretation of the Metric Learning Algorithms

When a person is making a movement, their behaviors are sequentially arranged in the brain in the following steps (Wise, 1985; Andersen and Cui, 2009): Step (1) Perception at a cognitive level by visual cortex, when they perceive the environment; Step (2) Planning and Decision-Making by prefrontal and posterior cortex deciding the movement and posterior and premotor cortex organizing movement sequences; and Step (3) Execution by the primary motor cortex to generate specific movement trajectories. Traditionally, BMIs have been mainly focused on (Step 3) decoding trajectories of movements from the primary motor cortex (M1) (Donoghue, 2002; Moritz et al., 2008; Lebedev and Nicolelis, 2017; Abiri et al., 2019). However, decoding intended goals, (Step 2), allows us to achieve more realistic BMIs with high accuracy and robustness (Srinivasan et al., 2006; Kim et al., 2019; Wang et al., 2020).

Numerous studies using intracortical recordings have shown that neuronal activities, firing rates, from other brain areas besides primary motor cortex (M1) can be associated with movement by presenting correlation of firing rates and movement procedures. For example, the lateral prefrontal cortex (LPFC), dorsal premotor cortex (PMd), and posterior parietal cortex [PPC, including lateral intraparietal area (LIP) and the parietal reach region (PRR)] are involved in movement planning and decision-making (Mountcastle et al., 1975; Wise, 1985; Miller and Cohen, 2001; Tanji and Hoshi, 2008; Desmurget et al., 2009). It has been found that although the posterior parietal cortex (PPC)'s function has been well-known for spatial attention and awareness (Hecaen, 1953; Colby and Goldberg, 1999), and the LPFC is mainly associated with voluntary attention (Tanji and Hoshi, 2008), it has been shown that action-related processes are highly linked to PPC, LPFC, PMd, and M1 (Goldman-Rakic, 1988; Andersen et al., 1990; Ojakangas et al., 2006; Schwedhelm et al., 2017). Moreover, Brodmann Area 5 in PPC has shown an involvement in reaching arm movement providing spatial information of the limb movement (Lacquaniti et al., 1995; Kalaska et al., 1997). In addition, (Ojakangas et al., 2006) presents movement intent decoding results on 4 direction and Go-No Go classification by comparing spike counts with respect to a specific class. Average classification accuracy were 38% on patient 1 and 2, and 47% on patient 3 in 4-direction classification, and patient 3 showed 73% average accuracy on the Go-No Go task. All neurons' activities were recorded from middle frontal gyrus. Although this study does not aim to decode pre-movement intention, it shows possibilities of using neural representations associated with movement planning and decision making.

In addition, Bereitschaftspotential (BP), or readiness potential, is a movement-related cortical potential that is observed prior to the movement onset. Observed initial slow negative potentials are reported 2 s before the movement onset (early BP) in the pre-supplementary motor area, and the steeper negative slope (late BP) have been reported before about 0.4 s prior to the movement onset (Shibasaki and Hallett, 2006; Schurger et al., 2021). In addition, several studies investigated possibilities of EEG pre-movement intention classification have been reported as described in Introduction Section (Novak et al., 2013; López-Larraz et al., 2014; Kim et al., 2019; Mohseni et al., 2020; Wang et al., 2020). Our study also confirms that the pre-movement intention can be distinguished between different tasks prior to the movement onset, using EEG. Furthermore, our study provides a unique addition to the EEG-based BMIs to classify EEG pre-movement intention using metric learning algorithms.

The explanation of which brain areas or neural patterns contribute to the movement planning is an important aspect to address when modeling BMIs. The problem of interpretability of machine learning algorithms has been receiving sharply increasing attention. The ability to explain how the algorithm decides its outputs can help build trust by practitioners and bring understanding that supports reliability. In addition, visual interpretation provides straightforward intuition about the data. This is a topic that has been receiving increasing attention in the deep learning communities where several methods for attribution have been proposed (Zeiler and Fergus, 2014; Selvaraju et al., 2017; Schulz et al., 2020). Interpretable methods have been considered to better understand brain activities using EEG. Recently, algorithms providing clinical interpretation have been introduced in seizure prediction in epilepsy (Uyttenhove, 2020; Pinto et al., 2022) and schizophrenia detection (Vázquez et al., 2021). In addition, Three-stage algorithm was introduced to classify two spatial tasks (Yi et al., 2022). The use of Layer-wise Relevance Propagation (LRP) has been introduced in EEG motor imagery classification (Sturm et al., 2016). Like Grad-CAM, LRP is an attribution method that provides interpretability on an instance basis. While this level of granularity may add to interpretability, it has been shown that, in the context of vision models (Ghorbani et al., 2019), attribution methods including LRP are brittle to adversarial attacks where small changes to the input may result in completely different attribution maps. In this case aggregate methods such as our *importance* or Common Spatial Pattern are still preferred.

The *importance* measure we propose is one possible but not the only way to assess the relevance of different brain areas in predicting movement. Other approaches based on attribution such as Grad-CAM (Selvaraju et al., 2017) can be considered. However, there is a main distinction between attribution methods and *importance*. Attribution methods usually work in a per data sample basis. Thus, to obtain the *importance* map, one must adapt the attribution methods to summarize the contributions of the input features for all data instances to the decision function. As more data becomes available, it would be interesting to explore alternative techniques that employ attribution to obtain the *importance* of brain areas.

We confirm that *importance* allows to visualize how much a given feature contributes to the decision of the neural decoder, and when key features are sorted based on this measure, explainable brain function can be visualized. We show that the learned metrics can capture the features that are relevant for classification irrespective of the structure of the classes, which offers the potential to highlight important information from the input that does not need to be linearly separable. Although this is still a preliminary result that is not yet sufficient to draw definite conclusions, our experiments support possible use of *importance* to visualize the contribution of features that can be related to brain function. In our experiments, metric learning did not provide any improvements in classification accuracy. However, we expect that with more experimental data, a non-linear classifier may provide better accuracy for which metric learning algorithms can provide a sensible way to track which input features are relevant (Goldberger et al., 2004).

Metric learning has been mainly considered as an integrative method to aid classification algorithms. Benefits of using metric learning in EEG classification have been reported in sleep stage classification (Phan et al., 2013), personal identification (Cai et al., 2015), epilepsy classification (Xue et al., 2020), and motor imagery classification (Alwasiti et al., 2020). The later, focuses on deep metric learning which learns a non-linear map for the metric that may not be easy to interpret in terms of the input features. In addition, the study suggests a different time-frequency representation of the signals, namely Stockwell transform that improves classification. However, it is novel and valuable to emphasize metric learning's interpretability on EEG data. Our proposed *importance* provides visual explanation of the data projections to maximize the inter class separations. Although this study provides meaningful interpretation of the EEG data, further implementation with various data sets is required. In addition, although we set the window size and time locations from our best reasoning and judgement, it is possible that different window sizes and time locations can provide more meaningful interpretation of the neurological representation of the pre-movement intentions and better classification performance. In addition, while the metric learning algorithms we explored are not the only ones available in the literature, we see the potential to apply the same *importance* measure to other metric learning algorithms, including (Weinberger et al., 2005; Davis et al., 2007; Jain et al., 2008; Taylor et al., 2011), that learn a linear map. Kulis (2013) provides a comprehensive review of various metric learning algorithms that can be integrated to this study.

In this study, we examined metric learning algorithm's interpretability in space, that is, which brain area provides more information for the intention classification. For the raw EEG, it is worth to note that interpretability can also be considered in time. When more data becomes available, it will be possible to inspect *importance* values for individual time instances. These can highlight which time intervals within the pre-movement or movement windows are more relevant for the classification. Moreover, other representation such as time frequency representation including the short time Fourier transform, wavelet transform, or the Stockwell transform employed in Alwasiti et al. (2020) can be used, as well. We plan to study this temporal aspect in future work.

### Available EEG Data and Feature Representations

Although several EEG data sets to investigate motor imagery are publicly available (Schalk et al., 2004; Blankertz et al., 2007; Luciw et al., 2014; Schirrmeister et al., 2017), the number of data sets that can be used to investigate pre-movement intentions are still limited. In addition, most of the experimental set ups do not allow to investigate freewill. Commonly used experimental set ups include stimulus-driven actions with externally decided targets based on light or color indicators. That is, the task or goal that the subject needs to accomplish is provided by the experimenter, beforehand by a visual or auditory cue. To the best of our knowledge, the data set we used here is the only publicly available EEG data set that allows to investigate freewill pre-movement intention. Due to the limited size of the data set, which contains only two individuals in three different day's recordings, our investigation cannot be generalized, yet. However, our study supports the potential of using raw EEG data, with only basic filtering to remove artifacts, to reliably classify pre-movement and movement intention. In addition, the use of frequency components has shown benefits for increasing the classification accuracy. Furthermore, metric learning algorithm that can select certain frequency components allow straightforward design of EEG-based BMIs. Thus, further investigation of the features beyond time or frequency representations could be beneficial to provide better interpretability of the metric learning algorithms and to bring high performance BMIs. Nevertheless, design of automatic selection of features would be essential to implement closed-loop BMIs.

It is important to highlight that non-linear SVM did not provide distinguishably superior classification accuracies. The classification accuracies obtained by linear and non-linear SVM do not show significant differences (within 1% differences on average). This may be due to the characteristics of the task conducted for these specific data sets, pressing two keys on the keyboard. Again, with the limited available data sets, our observations cannot be generalized. To further investigate behaviors of the metric learning and performances on the classifiers, our group has started collecting our own EEG data on reaching tasks that allow to investigate freewill pre-movement and movement intentions. Once a sufficient amount of data has been collected, we plan to apply the same methods to expand our study. It is expected that these data sets will allow us to better understand the selection of features, the timing of events related to pre movement intention, the role of metric learning algorithms, and the performance of linear vs. non-linear classifiers.

## Data Availability Statement

Publicly available datasets were analyzed in this study. The EEG datasets used for this study can be found in https://figshare.com/collections/A_large_electroencephalographic_motor_imagery_dataset_for_electroencephalographic_brain_computer_interfaces/3917698 (Kaya et al., 2018). In addition, the code used to generate all results in this study is available in the following GitHub repository: https://github.com/JihyeBae13/EEG_EGML. We modified MATLAB code that is publicly available at https://github.com/luisitobarcito/conditional-entropy-metric-learning to implement the metric learning algorithms, listed in our study.

## Ethics Statement

We used publicly available data set and their published study lists, all experiments were approved by the Ethics Committees of Toros University and Mersin University in the city of Mersin, Turkey (Kaya et al., 2018). The patients/participants provided their written informed consent to participate in that study.

## Author Contributions

JB conceived the presented idea. WP and LGSG performed data reconstruction and analysis and implemented the metric learning algorithms and the classifier. JB guided EEG processing and overall study design. LGSG mentored the metric learning application on WP and LGSG implemented the *importance* maps. JB and LGSG verified the analytical methods, results, and contributed on the manuscript writing. All authors contributed to the article and approved the submitted version.

## Funding

This work was partially supported by Electrical and Computer Engineering Undergraduate Research Fellowship and JB and LGSG Start Up Funds. These funds are provided by the Department of Electrical and Computer Engineering at the University of Kentucky. This material is based upon work supported by the Office of the Under Secretary of Defense for Research and Engineering under award number FA9550-21-1-0227.

## Conflict of Interest

The authors declare that the research was conducted in the absence of any commercial or financial relationships that could be construed as a potential conflict of interest.

## Publisher's Note

All claims expressed in this article are solely those of the authors and do not necessarily represent those of their affiliated organizations, or those of the publisher, the editors and the reviewers. Any product that may be evaluated in this article, or claim that may be made by its manufacturer, is not guaranteed or endorsed by the publisher.
