# Supplementary material for: Metric Learning in Freewill EEG Pre-Movement and Movement Intention Classification for Brain Machine Interfaces
Source: Front Hum Neurosci. 2022 Jul 1;16:902183. doi: 10.3389/fnhum.2022.902183 (PMC9283905; doi:10.3389/fnhum.2022.902183)
Supplement: Supplementary file 1 [file Data_Sheet_1.PDF]

Supplementary Material

1 Entropy Gap VS Matrix-based Mutual Information

In this section, we show how mutual information increases as a function of the Gaussian kernel parameter. In (Sanchez Giraldo, Rao et al. 2015), it is shown that for two normalized positive definite matrices $\boldsymbol{K}_1$ and $\boldsymbol{K}_2$ of size $n \times n$,

$$S_\alpha(\boldsymbol{K}_i) \leq S_\alpha(\boldsymbol{K}_1, \boldsymbol{K}_2) \text{ for } i = 1,2.$$

Let $(\boldsymbol{K}_1)_{ij} = \kappa_{\sigma_1}(\boldsymbol{y}_i, \boldsymbol{y}_j)$, $(\boldsymbol{K}_2)_{ij} = \kappa_{\sigma_2}(\boldsymbol{y}_i, \boldsymbol{y}_j)$, and $\boldsymbol{K}_\sigma = \boldsymbol{K}_1 \circ \boldsymbol{K}_2$, with

$$\kappa_h(\boldsymbol{y}_i, \boldsymbol{y}_j) = \exp\left(-\frac{\left(\boldsymbol{y}_i - \boldsymbol{y}_j\right)^2}{2h^2}\right).$$

Let $\sigma_1 = \frac{\sigma}{\sqrt{1-\lambda}}$ and $\sigma_2 = \frac{\sigma}{\sqrt{\lambda}}$, for any value of $\lambda \in (0,1)$, we have that

$$S_\alpha(\boldsymbol{K}_i) \leq S_\alpha(\boldsymbol{K}_1, \boldsymbol{K}_2) = S_\alpha(\boldsymbol{K}_\sigma),$$

$\sigma < \sigma_1$, and $\sigma < \sigma_2$. Based on this relation, we can see that the matrix entropy monotonically increases as the kernel width parameter σ becomes smaller. Note also that $S_\alpha(\boldsymbol{K}_\sigma) \leq \log n$, and $\lim_{\sigma \to 0} S_\alpha(\boldsymbol{K}_\sigma) = \log n$, if all samples of Y are different. For the matrix-mutual information between data and class labels given by

$$MI_\alpha(\boldsymbol{K}_L; \boldsymbol{K}_Y) = S_\alpha(\boldsymbol{K}_L) + S_\alpha(\boldsymbol{K}_Y) - S_\alpha(\boldsymbol{K}_L \circ \boldsymbol{K}_Y),$$

we have that $\lim_{\sigma \to 0} MI_\alpha(\boldsymbol{K}_L;\ \boldsymbol{K}_Y) = S_\alpha(\boldsymbol{K}_L)$, and $\lim_{\sigma \to \infty} MI_\alpha(\boldsymbol{K}_L;\ \boldsymbol{K}_Y) = 0$. On the other hand, for the entropy gap, we have $\lim_{\sigma \to \infty} E_\Pi[S_\alpha(L, Y_\Pi)] = \lim_{\sigma \to \infty} S_\alpha(\boldsymbol{K}_L \circ \boldsymbol{K}_Y) = S_\alpha(\boldsymbol{K}_L)$ and $\lim_{\sigma \to 0} E_\Pi[S_\alpha(L, Y_\Pi)] = \lim_{\sigma \to 0} S_\alpha(\boldsymbol{K}_L \circ \boldsymbol{K}_Y) = \log n$. Therefore, the entropy gap, will be larger for a value of σ that is not in any of the two extremes. Note that for the metric learning based on the maximization of the entropy gap, we can let the transformation matrix $\boldsymbol{A}$ absorb the values of the kernel size in the magnitude of its entries, which eliminates the traces constraint that was necessary in the conditional entropy metric learning algorithm.

2 Performance Measures

To provide a more detailed assessment of performance, we compute measures of sensitivity and predictive value for all reported cases on Figures 7 and 8. Below, we describe the measures of performance.

Given a confusion matrix (Supplementary Figure 1), where $PrdC_i\ from\ C_j$ denotes the number of points from class j that were classified as class i and n is the number of classes, we can define the following quantities:

- **Accuracy** is the percentage of data instances that were correctly classified from all data instances,

$$Accuracy = \frac{\sum_{i=1}^{n} PrdC_i\ from\ C_i}{\sum_{i=1}^{n}\sum_{k=1}^{n} PrdC_i\ from\ C_k} \times 100\%$$

- **Class *i* sensitivity** is the percentage of data instances from class i that are correctly classified,

$$Class\ i\ sensitivity = \frac{PrdC_i\ from\ C_i}{\sum_{k=1}^{n} PrdC_i\ from\ C_k} \times 100\%$$

- **Class *i* predictive value** is the percentage of correctly classified data instances from all data instances that were classified as class i,

$$Class\ i\ predictive\ value = \frac{PrdC_i\ from\ C_i}{\sum_{k=1}^{n} PrdC_k\ from\ C_i} \times 100\%$$

Like accuracy, higher class sensitivity and class predictive value mean better performance.

Supplementary Tables 1 through 4 display the different performance measures for all combinations of subject, feature, metric learning algorithm, and classifier. Note that Supplementary Tables 1 and 2 match to Figures 7 and 8 (a) through (c), respectively. Also, Supplementary Table 3 and 4 correspond to Figures 7 and 8 (d) through (f).

Supplementary Figure 1. Conceptual Confusion Matrix

	**Predicted C_1**	**Predicted C_2**	⋯	**Predicted C_n**
**True C_1**	$PrdC_1$ from C_1	$PrdC_2$ from C_1	⋯	$PrdC_n$ from C_1
**True C_2**	$PrdC_1$ from C_2	$PrdC_2$ from C_2	⋯	$PrdC_n$ from C_2
⋮	⋮	⋮	⋱	⋮
**True C_n**	$PrdC_1$ from C_n	$PrdC_2$ from C_n	⋯	$PrdC_n$ from C_n

Supplementary Table 1. Detailed performance measures for the pre-movement intention for linear SVM.

Data		Learning Method	Accuracy	Class 1 sensitivity	Class 1 predictive value	Class 2 sensitivity	Class 2 predictive value
Feature1-A	B	Euclidean	60.62(8.98)	62.53(9.91)	60.85(9.59)	59.36(11.06)	60.96(12.65)
		CEML	57.50(10.13)	59.51(10.37)	58.43(11.43)	56.68(13.84)	57.83(12.72)
		NCA	58.86(7.85)	60.86(10.32)	58.93(9.68)	57.74(8.33)	59.60(11.78)
		EGML	57.64(6.89)	59.49(7.89)	58.06(9.58)	56.30(10.74)	57.87(9.95)
	C1	Euclidean	73.68(4.77)	74.33(8.23)	73.62(7.56)	73.34(7.43)	74.02(7.95)
		CEML	67.59(5.13)	67.00(10.90)	68.03(8.07)	68.69(6.27)	67.61(8.61)
		NCA	69.18(7.05)	69.13(11.87)	68.60(8.94)	68.70(7.32)	69.39(7.63)
		EGML	72.39(3.84)	72.09(8.56)	72.66(5.73)	72.92(4.17)	72.26(7.66)
	C2	Euclidean	66.57(5.18)	64.73(8.15)	67.55(7.25)	68.34(9.55)	65.71(8.79)
		CEML	64.29(3.50)	63.76(4.75)	64.11(4.90)	64.01(6.87)	63.63(7.44)
		NCA	67.43(5.90)	66.31(6.68)	67.81(8.86)	67.95(11.41)	66.60(7.64)
		EGML	66.86(5.55)	68.31(6.17)	66.71(7.28)	65.42(9.57)	66.84(10.01)
Feature1-B	B	Euclidean	58.73(5.74)	58.80(7.48)	58.93(6.53)	58.81(7.17)	58.79(7.17)
		CEML	58.73(5.03)	58.34(7.79)	59.18(5.53)	59.36(8.17)	58.77(6.89)
		NCA	59.40(5.70)	59.57(6.29)	59.82(6.93)	59.40(8.85)	59.31(6.40)
		EGML	57.63(5.81)	57.87(8.68)	58.00(7.83)	57.64(9.59)	57.84(7.07)
	C1	Euclidean	79.94(5.15)	78.38(6.42)	80.67(7.46)	81.45(6.99)	79.00(6.14)

		CEML	59.85(7.22)	61.80(7.93)	59.84(11.45)	57.81(9.93)	59.73(7.60)
		NCA	74.41(3.49)	75.73(6.92)	73.72(6.87)	73.18(4.14)	74.87(6.84)
		EGML	78.77(5.95)	76.75(5.98)	79.95(8.13)	80.71(8.54)	77.29(6.51)
	**C2**	Euclidean	72.71(3.33)	70.06(8.63)	74.11(6.30)	75.29(7.25)	71.75(8.56)
		CEML	59.43(5.92)	59.17(9.61)	59.71(5.25)	59.87(8.52)	59.25(10.94)
		NCA	67.57(5.30)	67.79(10.04)	68.32(9.27)	67.69(12.96)	67.68(10.16)
		EGML	70.57(6.07)	73.11(8.69)	69.93(8.63)	68.70(9.02)	71.49(10.95)
**Feature2**	**B**	Euclidean	59.26(3.97)	56.74(9.98)	59.37(7.81)	61.33(7.40)	58.84(7.05)
		CEML	56.70(3.83)	52.80(10.68)	56.75(7.48)	59.88(8.53)	56.08(6.66)
		NCA	58.45(4.38)	57.05(9.54)	58.69(10.91)	59.68(8.85)	58.44(4.01)
		EGML	55.62(4.15)	54.28(5.46)	56.15(9.43)	57.20(8.98)	55.37(6.19)
	**C1**	Euclidean	73.26(7.10)	72.03(7.95)	74.04(8.81)	74.61(8.99)	72.28(9.46)
		CEML	71.07(6.67)	68.51(8.32)	72.39(7.57)	73.23(8.46)	69.45(9.65)
		NCA	70.05(7.26)	71.04(8.94)	69.59(7.38)	68.54(7.90)	69.82(9.88)
		EGML	70.62(7.25)	68.26(8.22)	71.53(9.83)	73.05(8.04)	69.49(8.01)
	**C2**	Euclidean	66.57(5.23)	64.45(10.26)	66.60(7.81)	68.27(5.02)	66.33(6.08)
		CEML	67.14(5.04)	64.71(10.30)	68.07(5.87)	69.57(6.73)	66.72(8.35)
		NCA	66.00(6.35)	67.73(10.15)	65.17(7.44)	64.32(6.09)	67.05(9.08)
		EGML	66.00(6.99)	63.19(7.98)	66.97(7.50)	68.58(9.36)	64.87(8.57)

Supplementary Table 2. Detailed performance measures for pre-movement intention for nonlinear SVM.

Data		Learning Method	Accuracy	Class 1 sensitivity	Class 1 predictive value	Class 2 sensitivity	Class 2 predictive value
Feature1-A	B	Euclidean	59.94(6.87)	61.25(10.85)	60.20(8.28)	59.49(8.96)	60.67(11.63)
		CEML	56.15(7.81)	56.63(10.81)	57.25(11.11)	57.32(12.30)	56.75(9.45)
		NCA	59.53(6.81)	60.68(5.97)	60.45(10.70)	59.37(11.66)	59.72(8.50)
		EGML	56.42(6.96)	60.24(8.66)	56.26(8.71)	52.67(9.83)	56.85(10.92)
	C1	Euclidean	72.23(3.21)	72.45(9.63)	72.46(6.03)	71.92(7.74)	72.58(7.46)
		CEML	66.71(3.95)	70.76(10.69)	65.63(5.83)	62.47(8.06)	68.40(9.03)
		NCA	69.62(5.20)	70.41(11.19)	68.77(7.02)	68.49(3.53)	70.41(7.36)
		EGML	72.39(2.87)	71.96(6.83)	72.63(5.45)	72.88(3.63)	72.10(6.12)
	C2	Euclidean	67.57(4.57)	66.70(7.33)	68.05(4.86)	68.51(6.50)	66.97(9.63)
		CEML	63.86(5.00)	62.19(7.89)	64.40(8.19)	65.15(9.57)	63.17(8.45)
		NCA	65.14(2.45)	63.10(3.99)	65.58(7.19)	67.23(3.80)	64.42(6.12)
		EGML	64.71(5.35)	64.26(8.96)	64.61(7.71)	64.92(7.53)	64.53(9.07)
Feature1-B	B	Euclidean	56.69(6.29)	56.88(9.64)	56.67(7.67)	56.75(6.79)	56.96(7.40)
		CEML	57.78(6.75)	58.27(9.17)	58.35(8.92)	57.47(12.02)	57.75(6.14)
		NCA	58.87(5.86)	60.31(4.32)	59.07(6.84)	57.62(9.11)	58.81(6.40)
		EGML	56.96(5.26)	58.54(8.68)	56.79(6.87)	55.42(7.34)	57.42(5.85)
	C1	Euclidean	78.19(6.73)	80.31(8.06)	77.07(9.03)	76.19(9.55)	79.20(8.47)

		CEML	62.92(8.45)	65.34(13.67)	61.65(11.20)	58.66(13.48)	62.98(10.08)
		NCA	67.86(5.16)	69.73(6.40)	67.54(8.13)	66.10(7.27)	68.18(7.84)
		EGML	78.34(5.31)	76.44(6.52)	79.67(7.25)	80.47(7.10)	76.85(6.87)
	**C2**	Euclidean	68.86(4.30)	68.46(4.68)	69.30(10.28)	69.92(8.79)	68.55(7.21)
		CEML	58.57(6.14)	59.78(9.37)	58.33(8.29)	57.83(5.55)	59.17(10.01)
		NCA	62.00(4.05)	61.05(7.89)	62.66(6.98)	63.40(8.85)	61.71(9.35)
		EGML	69.14(7.13)	68.33(10.36)	70.21(9.39)	70.60(10.11)	68.89(11.69)
**Feature2**	**B**	Euclidean	57.64(4.66)	58.60(7.57)	57.57(9.24)	56.15(11.30)	57.23(7.58)
		CEML	55.48(3.64)	53.46(10.35)	55.80(7.85)	57.52(9.31)	55.43(7.23)
		NCA	59.40(4.08)	59.24(9.04)	59.64(9.80)	59.62(9.83)	59.49(6.09)
		EGML	56.43(4.52)	55.70(7.01)	57.00(8.22)	57.74(8.67)	56.45(7.97)
	**C1**	Euclidean	72.81(6.01)	73.00(5.81)	72.75(7.72)	72.45(7.70)	72.37(7.53)
		CEML	66.27(6.45)	68.42(10.01)	65.87(8.27)	64.44(7.58)	66.80(10.40)
		NCA	68.89(6.81)	70.36(8.31)	68.74(8.26)	67.43(9.17)	68.90(10.01)
		EGML	71.07(8.55)	70.27(9.41)	71.57(9.35)	71.74(10.73)	70.08(11.01)
	**C2**	Euclidean	69.00(3.37)	66.04(7.17)	69.84(6.34)	71.71(4.99)	68.13(5.52)
		CEML	65.29(4.76)	65.33(6.79)	65.16(7.19)	65.10(6.45)	65.33(6.41)
		NCA	66.43(5.01)	67.85(8.17)	66.16(6.79)	65.16(9.24)	66.90(7.45)
		EGML	67.71(3.51)	66.98(4.66)	67.66(5.40)	68.10(4.57)	67.30(5.62)

Supplementary Table 3. Detailed performance measures for movement intention for linear SVM.

Data		Learning Method	Accuracy	Class 1 sensitivity	Class 1 predictive value	Class 2 sensitivity	Class 2 predictive value
Feature1-A	B	Euclidean	65.63(6.75)	66.83(9.19)	65.63(4.34)	64.89(6.53)	65.75(12.48)
		CEML	68.62(5.42)	68.35(9.41)	68.59(5.95)	68.99(5.27)	68.59(9.76)
		NCA	70.50(3.62)	72.53(8.30)	70.15(5.19)	69.18(5.39)	71.42(9.75)
		EGML	66.30(5.74)	66.92(8.85)	66.15(7.05)	66.22(4.17)	66.63(9.87)
	C1	Euclidean	84.46(4.29)	84.15(6.76)	84.85(6.10)	84.72(6.28)	84.07(7.50)
		CEML	79.23(5.63)	80.54(7.20)	78.93(5.14)	78.06(6.23)	79.25(9.76)
		NCA	80.39(5.04)	79.78(5.30)	81.30(6.93)	81.08(7.07)	79.34(8.22)
		EGML	78.22(13.26)	79.48(8.78)	78.94(13.69)	77.41(18.83)	77.00(15.09)
	C2	Euclidean	87.71(3.58)	88.03(5.61)	87.62(5.36)	87.89(4.22)	87.66(6.87)
		CEML	82.29(4.32)	82.28(6.73)	82.31(5.82)	82.22(5.88)	82.31(6.73)
		NCA	83.57(5.44)	86.03(5.47)	82.42(6.95)	81.39(8.24)	84.78(7.40)
		EGML	85.57(3.78)	88.43(7.03)	84.08(5.24)	83.06(6.15)	87.36(8.05)
Feature1-B	B	Euclidean	69.42(6.01)	71.10(7.56)	68.85(7.13)	67.93(7.17)	70.21(6.69)
		CEML	65.78(5.55)	65.33(9.93)	66.21(4.36)	66.65(5.61)	65.88(9.02)
		NCA	69.96(5.63)	70.10(7.37)	69.94(7.15)	69.73(7.73)	70.20(6.24)
		EGML	69.83(5.62)	71.61(6.36)	69.13(5.66)	67.96(6.27)	70.48(6.82)
	C1	Euclidean	90.84(3.09)	90.75(5.72)	91.05(5.48)	91.09(5.37)	90.90(4.99)

		CEML	78.63(4.71)	78.14(8.49)	79.82(7.29)	79.63(7.50)	78.24(8.99)
		NCA	87.49(4.49)	86.88(8.98)	88.05(4.70)	87.96(5.35)	87.59(7.48)
		EGML	91.27(2.69)	91.57(6.45)	91.10(4.66)	91.13(4.72)	91.75(5.33)
	**C2**	Euclidean	88.14(4.62)	86.78(8.60)	88.77(6.42)	89.43(5.74)	87.96(5.72)
		CEML	73.29(13.06)	73.84(12.62)	73.22(16.70)	72.82(16.26)	73.96(11.37)
		NCA	85.14(5.68)	85.05(9.35)	84.51(7.62)	85.31(5.60)	85.96(6.72)
		EGML	89.14(3.31)	87.92(6.95)	90.07(6.22)	90.54(5.52)	88.59(5.15)
**Feature2**	**B**	Euclidean	68.34(3.13)	68.57(5.08)	68.37(6.12)	68.00(6.87)	68.30(4.75)
		CEML	69.55(4.00)	71.27(4.69)	69.41(8.30)	67.76(9.24)	70.05(4.66)
		NCA	68.48(2.79)	69.26(4.93)	68.46(6.70)	67.99(6.17)	68.78(4.93)
		EGML	65.09(4.14)	67.21(8.77)	64.92(5.98)	63.63(6.80)	66.09(8.61)
	**C1**	Euclidean	87.50(2.67)	87.38(2.92)	87.83(5.25)	87.83(5.14)	87.05(4.09)
		CEML	80.23(3.68)	82.42(4.57)	79.36(6.28)	78.08(7.27)	81.23(5.23)
		NCA	82.71(1.82)	84.70(5.38)	81.91(5.23)	80.52(6.17)	83.86(5.16)
		EGML	85.17(4.23)	84.84(5.69)	85.95(6.70)	85.69(6.82)	84.62(6.35)
	**C2**	Euclidean	86.57(3.51)	87.34(5.58)	85.80(4.51)	85.62(4.14)	87.21(5.58)
		CEML	83.57(4.48)	82.60(7.47)	84.05(6.79)	84.86(4.92)	83.10(6.59)
		NCA	83.29(3.37)	82.19(6.63)	83.79(6.51)	84.51(5.37)	82.78(5.47)
		EGML	86.14(4.15)	87.07(5.38)	85.37(5.21)	84.95(5.28)	86.75(5.35)

Supplementary Table 4. Detailed performance measures for movement intention for nonlinear SVM.

Data		Learning Method	Accuracy	Class 1 sensitivity	Class 1 predictive value	Class 2 sensitivity	Class 2 predictive value
Feature1-A	B	Euclidean	69.96(5.22)	70.49(6.96)	69.97(6.83)	69.49(7.97)	69.84(8.99)
		CEML	68.07(2.78)	70.98(7.14)	67.36(6.18)	65.74(5.49)	69.24(9.01)
		NCA	70.64(4.68)	71.62(9.16)	70.45(5.37)	70.17(4.61)	71.11(10.68)
		EGML	66.70(5.04)	66.34(6.70)	66.52(7.85)	66.91(6.56)	66.37(7.16)
	C1	Euclidean	84.45(3.35)	84.11(6.84)	85.20(5.47)	85.06(5.71)	83.87(7.79)
		CEML	78.50(4.10)	80.36(5.62)	77.71(6.76)	77.03(4.87)	79.11(7.91)
		NCA	80.25(5.43)	80.50(7.75)	80.25(6.05)	79.89(5.89)	80.08(9.26)
		EGML	84.02(5.00)	83.39(7.34)	84.79(7.06)	85.40(5.55)	83.17(8.66)
	C2	Euclidean	87.29(2.56)	88.19(5.16)	87.04(5.17)	87.05(4.70)	87.52(6.72)
		CEML	81.71(5.03)	85.26(5.45)	80.20(8.15)	78.95(8.76)	83.77(6.74)
		NCA	82.43(4.42)	83.27(6.81)	82.18(6.03)	82.17(5.97)	82.69(7.87)
		EGML	84.14(3.84)	86.53(8.00)	82.95(4.96)	82.35(5.04)	85.58(8.93)
Feature1-B	B	Euclidean	67.39(5.92)	66.73(8.13)	67.73(6.86)	68.01(7.27)	67.26(6.93)
		CEML	64.69(3.22)	68.26(6.80)	63.88(4.22)	61.28(5.66)	65.96(6.28)
		NCA	66.99(4.52)	68.56(9.45)	66.46(3.78)	65.52(4.24)	67.85(8.04)
		EGML	68.06(5.99)	68.27(8.50)	67.96(7.46)	67.90(7.24)	68.40(6.22)
	C1	Euclidean	87.93(4.35)	88.01(7.90)	88.10(7.26)	88.12(7.65)	88.55(6.32)

		CEML	79.21(2.91)	79.78(6.77)	79.31(6.17)	78.79(6.92)	79.55(5.87)
		NCA	80.09(3.93)	79.98(10.04)	80.94(5.97)	80.44(8.13)	80.32(8.97)
		EGML	91.56(2.08)	92.15(5.74)	91.12(4.13)	91.06(3.88)	92.21(4.79)
	**C2**	Euclidean	87.29(5.32)	87.06(8.21)	87.48(9.92)	87.60(8.70)	88.04(5.91)
		CEML	76.43(8.89)	76.54(10.63)	77.03(11.66)	76.72(11.66)	76.67(9.94)
		NCA	79.71(5.03)	80.35(6.77)	79.38(9.56)	79.23(8.04)	80.45(5.30)
		EGML	90.14(3.46)	89.24(5.98)	90.47(5.69)	91.16(4.31)	89.85(4.56)
**Feature2**	**B**	Euclidean	67.26(3.54)	69.73(6.28)	66.64(5.40)	64.59(7.66)	68.06(6.21)
		CEML	66.44(5.08)	68.21(4.25)	66.17(6.31)	64.54(8.24)	66.59(7.52)
		NCA	68.88(3.28)	71.73(5.60)	67.79(5.85)	65.78(6.18)	70.04(4.35)
		EGML	66.71(6.30)	67.80(10.20)	66.83(8.14)	66.06(9.66)	67.48(8.31)
	**C1**	Euclidean	85.47(3.38)	86.04(4.84)	85.16(5.30)	84.78(5.64)	85.77(4.42)
		CEML	79.50(5.19)	82.39(7.14)	77.95(7.22)	76.88(6.51)	81.20(7.12)
		NCA	82.12(4.44)	82.64(7.85)	82.08(7.38)	81.86(7.13)	82.56(7.25)
		EGML	84.01(4.13)	83.94(5.15)	84.39(6.05)	84.27(5.87)	83.64(6.11)
	**C2**	Euclidean	87.00(4.01)	88.14(6.17)	85.75(6.46)	86.07(4.51)	88.22(5.78)
		CEML	81.57(3.53)	81.99(6.93)	81.55(6.63)	81.11(7.32)	82.08(6.58)
		NCA	81.86(4.72)	82.68(6.95)	81.36(8.80)	81.73(7.29)	82.59(6.83)
		EGML	85.86(5.49)	85.39(6.86)	86.41(6.76)	85.98(7.20)	85.43(6.51)

3 Statistical Analysis on the Performance

To compare the test accuracies among all the different implementation of metric learning, we performed paired t-test on the classification accuracies for each one of the test-folds. In short, each algorithm was tested on the same test folds. The paired t-test computes the difference between the accuracies of two algorithms and test the null hypothesis of whether these differences are zero. The tables below contain the p-values for each pair of algorithms on a different combination of subject and feature for pre-movement intention (Supplementary Tables 5 through 13). Bold numbers, both black and red, correspond to the cases where the null hypothesis is rejected with 5% significance level. The red color means the method on the column has higher accuracy (performs better) than the method on the row and the bold black the opposite. As we can see for all subject and feature combinations, the comparison between the Euclidean baseline and EGML, consistently show no statistically significant difference between the test accuracies of both methods, whereas other methods vary across subjects and features.

Supplementary Table 5. p-values for paired t-test on the cross-validation test accuracies for the pre-movement intention classification with **Feature1-A** and Subject B.

		**Linear**			**Nonlinear**			
		CEML	NCA	EGML	Euclidean	CEML	NCA	EGML
**Linear**	Euclidean	0.3436	0.0509	0.1552	0.6942	0.2131	0.6670	0.0574
	CEML		0.6218	0.9486	0.4038	0.7069	0.4368	0.5818
	NCA			0.3981	0.4100	0.4012	0.7192	0.1042
	EGML				0.2169	0.5081	0.2469	0.2858
**Nonlinear**	Euclidean					0.2640	0.7717	**0.0176**
	CEML						0.2404	0.9215
	NCA							**0.0094**

Supplementary Table 6. p-values for paired t-test on the cross-validation test accuracies for the pre-movement intention classification with **Feature1-A** and Subject C Data 1.

		**Linear**			**Nonlinear**			
		CEML	NCA	EGML	Euclidean	CEML	NCA	EGML
**Linear**	Euclidean	**0.0039**	**0.0283**	0.4331	0.1988	**0.0031**	**0.0219**	0.4291
	CEML		0.5471	**0.0013**	**0.0015**	0.5698	0.1515	**0.0050**
	NCA			0.1381	0.1498	0.2996	0.8067	0.1711
	EGML				0.8845	**0.0016**	**0.0347**	1.0000
**Nonlinear**	Euclidean					**0.0014**	0.0511	0.8875
	CEML						0.0597	**0.0005**
	NCA							**0.0404**

Supplementary Table 7. p-values for paired t-test on the cross-validation test accuracies for the pre-movement intention classification with **Feature1-A** and Subject C Data 2.

		Linear			Nonlinear			
		CEML	NCA	EGML	Euclidean	CEML	NCA	EGML
**Linear**	Euclidean	0.0954	0.5414	0.8321	0.4716	0.0882	0.3582	0.2116
	CEML		0.1111	0.1619	0.0511	0.8249	0.5203	0.8056
	NCA			0.7109	0.9450	**0.0327**	0.1679	0.1737
	EGML				0.7080	**0.0399**	0.3399	0.0569
**Nonlinear**	Euclidean					**0.0346**	0.1379	0.1582
	CEML						0.3371	0.6221
	NCA							0.8086

Supplementary Table 8. p-values for paired t-test on the cross-validation test accuracies for the pre-movement intention classification with **Feature1-B** and Subject B.

		**Linear**			**Nonlinear**			
		CEML	NCA	EGML	Euclidean	CEML	NCA	EGML
**Linear**	Euclidean	0.9978	0.7216	0.5859	0.1765	0.6796	0.9498	0.5531
	CEML		0.6869	0.6057	0.2625	0.6746	0.9233	0.5257
	NCA			0.2881	0.2617	0.4551	0.7514	0.3257
	EGML				0.5407	0.9560	0.6006	0.7790
**Nonlinear**	Euclidean					0.6896	0.4106	0.9297
	CEML						0.5532	0.7886
	NCA							0.4967

Supplementary Table 9. p-values for paired t-test on the cross-validation test accuracies for the pre-movement intention classification with **Feature1-B** and Subject C Data 1.

		**Linear**			**Nonlinear**			
		CEML	NCA	EGML	Euclidean	CEML	NCA	EGML
**Linear**	Euclidean	**0.0000**	**0.0239**	0.5700	0.3763	**0.0003**	**0.0016**	0.3448
	CEML		**0.0000**	**0.0001**	**0.0001**	0.1773	**0.0014**	**0.0000**
	NCA			**0.0300**	0.0972	**0.0012**	**0.0015**	**0.0244**
	EGML				0.7576	**0.0010**	**0.0010**	0.7980
**Nonlinear**	Euclidean					**0.0001**	**0.0024**	0.9048
	CEML						0.0978	**0.0001**
	NCA							**0.0008**

Supplementary Table 10. p-values for paired t-test on the cross-validation test accuracies for the pre-movement intention classification with **Feature1-B** and Subject C Data 2.

		Linear			Nonlinear			
		CEML	NCA	EGML	Euclidean	CEML	NCA	EGML
**Linear**	Euclidean	**0.0000**	**0.0345**	0.3270	**0.0257**	**0.0002**	**0.0001**	0.1316
	CEML		**0.0011**	**0.0007**	**0.0019**	0.7469	0.1436	**0.0102**
	NCA			**0.0376**	0.4500	**0.0064**	**0.0156**	0.5233
	EGML				0.2339	**0.0049**	**0.0032**	0.5779
**Nonlinear**	Euclidean					**0.0047**	**0.0065**	0.9047
	CEML						0.1169	**0.0039**
	NCA							**0.0209**

Supplementary Table 11. p-values for paired t-test on the cross-validation test accuracies for the pre-movement intention classification with **Feature2** and Subject B.

		Linear			Nonlinear			
		CEML	NCA	EGML	Euclidean	CEML	NCA	EGML
**Linear**	Euclidean	0.1819	0.6522	0.0515	0.3245	**0.0281**	0.9322	0.2021
	CEML		0.2187	0.6128	0.6264	0.5799	0.0793	0.9058
	NCA			0.1358	0.6565	0.1679	0.3417	0.3673
	EGML				0.2710	0.9161	0.0607	0.4816
**Nonlinear**	Euclidean					0.2922	0.2961	0.4411
	CEML						0.0851	0.5211
	NCA							0.2160

Supplementary Table 12. p-values for paired t-test on the cross-validation test accuracies for the pre-movement intention classification with **Feature2** and Subject C Data 1.

		Linear			Nonlinear			
		CEML	NCA	EGML	Euclidean	CEML	NCA	EGML
**Linear**	Euclidean	0.2262	0.0772	0.1634	0.7321	**0.0073**	**0.0426**	0.1954
	CEML		0.4387	0.8432	0.1261	**0.0067**	0.0712	1.0000
	NCA			0.7737	**0.0199**	**0.0023**	0.2589	0.6031
	EGML				0.2325	0.0626	0.3946	0.7431
**Nonlinear**	Euclidean					**0.0002**	**0.0083**	0.3819
	CEML						**0.0211**	0.0595
	NCA							0.3571

Supplementary Table 13. p-values for paired t-test on the cross-validation test accuracies for the pre-movement intention classification with **Feature2** and Subject C Data 2.

		**Linear**			**Nonlinear**			
		CEML	NCA	EGML	Euclidean	CEML	NCA	EGML
**Linear**	Euclidean	0.8082	0.7655	0.7851	0.1519	0.5926	0.9447	0.5871
	CEML		0.4664	0.5185	0.1217	0.0833	0.5911	0.6370
	NCA			1.0000	**0.0422**	0.6942	0.8100	0.3572
	EGML				0.1485	0.7477	0.8394	0.4498
**Nonlinear**	Euclidean					**0.0043**	0.0744	0.2146
	CEML						0.5338	0.1013
	NCA							0.1341
